# Supplementary material for: Randomised controlled trial on the effect of internet-delivered computerised cognitive–behavioural therapy on patients with insomnia who remain symptomatic following hypnotics: a study protocol
Source: BMJ Open. 2018 Jan 30;8(1):e018220. doi: 10.1136/bmjopen-2017-018220 (PMC5829590; doi:10.1136/bmjopen-2017-018220)
Supplement: Author's manuscript [file bmjopen-2017-018220.draft_revisions.pdf]

BMJ Open

BMJ Open is committed to open peer review. As part of this commitment we make the peer review history of every article we publish publicly available.

When an article is published we post the peer reviewers' comments and the authors' responses online. We also post the versions of the paper that were used during peer review. These are the versions that the peer review comments apply to.

The versions of the paper that follow are the versions that were submitted during the peer review process. They are not the versions of record or the final published versions. They should not be cited or distributed as the published version of this manuscript.

BMJ Open is an open access journal and the full, final, typeset and author-corrected version of record of the manuscript is available on our site with no access controls, subscription charges or pay-per-view fees (http://bmjopen.bmj.com).

If you have any questions on BMJ Open's open peer review process please email editorial.bmjopen@bmj.com

BMJ Open

Study protocol for a pilot randomized controlled trial of internet-delivered computerized cognitive behavioral therapy for insomnia who remain symptomatic following hypnotics

Journal:	*BMJ Open*
Manuscript ID	bmjopen-2017-018220
Article Type:	Protocol
Date Submitted by the Author:	11-Aug-2017
Complete List of Authors:	Sato, Daisuke; Chiba University Graduate School of Medicine, Departments of Cognitive Behavioral Physiology; Chiba Prefectural University of Health Sciences, Rehabilitation Sciences<br>Yoshinaga, Naoki; Miyazaki Daigaku, Organization for Promotion of Tenure Track<br>Nagai, Eichi; Chiba University Hospital, Clinical Research Center<br>Hanaoka, Hideki; Chiba University Hospital, Clinical Research Center<br>Sato, Yasunori; Chiba University Graduate School of Medicine, Department of Global Clinical Research / Biostatistics<br>Shimizu, Eiji; Chiba University, Department of Cognitive Behavioral Physiology, Graduate School of Medicine; Chiba University, Research Center for Child Mental Development
**Primary Subject Heading**:	Mental health
Secondary Subject Heading:	Evidence based practice, Patient-centred medicine
Keywords:	Cognitive therapy, Insomnia, Randomised controlled trial, Internet

SCHOLARONE™
Manuscripts

Study protocol for a pilot randomized controlled trial of internet-delivered computerized cognitive behavioral therapy for insomnia who remain symptomatic following hypnotics

Daisuke Sato,[1, 2] Naoki Yoshinaga,[3] Eichi Nagai,[4] Hideki Hanaoka,[4] Yasunori Sato,[5] Eiji Shimizu[1, 6, 7]

1) Departments of Cognitive Behavioral Physiology, Chiba University Graduate School of Medicine, Chiba, Japan
2) Department of Rehabilitation Sciences, Faculty of Health Care Sciences, Chiba Prefectural University of Health Sciences, Chiba, Japan
3) Organization for Promotion of Tenure Track, University of Miyazaki, Miyazaki, Japan
4) Clinical Research Center, Chiba University Hospital, Chiba, Japan
5) Department of Global Clinical Research / Biostatistics, Graduate School of Medicine, Chiba University, Chiba, Japan
6) Research Center for Child Mental Development, Chiba University, Chiba, Japan
7) Cognitive Behavioral Therapy Center, Chiba University Hospital, Chiba, Japan

Corresponding author

Daisuke Sato,[1, 2]

1) Departments of Cognitive Behavioral Physiology, Chiba University Graduate School of Medicine, Chiba, Japan
2) Department of Rehabilitation Sciences, Faculty of Health Care Sciences, Chiba Prefectural University of Health Sciences, Chiba, Japan

Departments of Cognitive Behavioral Physiology, Chiba University Graduate School of Medicine, Chiba, Japan
1-8-1 Inohana, Chuo-ku, Chiba 260-8670, Japan
Tel: (+81) 43-226-2027, Fax: (+81) 43-226-2028
E-mail: daisu.sato@gmail.com

Keywords: Cognitive therapy, Insomnia, Randomised controlled trial, Internet

Word count: 3007

Number of tables and figures: 46

For peer review only - http://bmjopen.bmj.com/site/about/guidelines.xhtml

ABSTRACT

Introduction: Insomnia has severe consequences for health. Primary care doctors commonly provide hypnotics in Japan, which is far from optimal. The recommended treatment for insomnia is cognitive behavioral therapy (CBT). Access to trained therapists, however, is limited. Rather than face-to-face CBT, several researchers have studied internet-delivered computerized CBT (ICBT). This paper describes the study protocol for a pilot randomized controlled trial (RCT) to evaluate effectiveness and feasibility of our newly developed 5-step ICBT as an adjunct to usual care (UC) compared with UC alone for patients with insomnia who remain symptomatic following hypnotics.

Methods and analysis: This proposed exploratory RCT comprises two parallel groups (ICBT + UC, and UC alone) consisting of 15 participants each (n = 30) diagnosed with insomnia who remain symptomatic after pharmacotherapy. We aim to evaluate the effectiveness of 6 intervention weeks. The primary outcome of insomnia severity will be the Pittsburgh Sleep Quality Index (PSQI) at week 6. Secondary outcomes will be: sleep onset latency, total sleep time, and sleep efficiency extracted from PSQI, current feeling of refreshment and soundness of sleep measured by visual analog scale, number of awakenings, anxiety by Hospital Anxiety and Depression Scale, depression by Center for Epidemiologic Studies Depression Scale, and quality of life by Euro Qol-5D. All measures will be assessed at weeks 0 (baseline), 6 (post-intervention), and 12 (follow-up); the intention-to-treat principle will be followed. Statistical analysis plan has been developed so that field materials can be appropriately designed.

Ethics and dissemination: This study will be conducted at the academic outpatient clinic of Chiba University Hospital, Japan. Ethics approval was granted by the Institutional Review Board of Chiba University Hospital. All participants will be required to provide written informed consent. The trial will be implemented and reported in accordance with CONSORT recommendations.

Clinical Trial Registration Number: UMIN000021509

For peer review only - http://bmjopen.bmj.com/site/about/guidelines.xhtml

ARTICLE SUMMARY

Article focus

- Pharmacotherapy, including non-benzodiazepines, benzodiazepines, melatonin receptor agonists, orexin receptor antagonists and antidepressants, is the first-choice treatment of insomnia in clinical practice; however, some patients were less satisfied with their medications. Patients with insomnia who remain symptomatic following pharmacotherapy prefer easy-access therapy, such as internet-delivered computerized cognitive behavioral therapy (ICBT). In this pilot randomized controlled trial (RCT), our newly developed 5-step ICBT for insomnia intervention will be examined to determine its effectiveness and feasibility for the treatment of patients with insomnia resistant to pharmacotherapy.

Key messages

- A pilot RCT protocol is outlined for the evaluation of clinical effectiveness and feasibility of our newly developed internet-delivered computerized cognitive behavior therapy as a next-step strategy, when administered concomitantly with pharmacotherapy.

Strengths and limitations of this study

- This is the first pilot RCT to focus on ICBT as a therapeutic option for insomnia patients who remain symptomatic following hypnotics.
- This pilot study reflects good clinical practice, and its results will contribute to the development of second-line treatments and establish future treatment algorithms.
- A limitation of this study is that we will be unable to elucidate specific effects of the ICBT program because a psychological placebo group to control for nonspecific factors will not be employed and sleep estimates will not be based on objective measures such as polysomnography or actigraphy.

For peer review only - http://bmjopen.bmj.com/site/about/guidelines.xhtml

INTRODUCTION

Insomnia is characterized by inability to fall asleep or awakening too early in the morning or during the night, resulting in non-restorative sleep and decreased daytime functioning. Insomnia comprises a symptomatic state of difficulty sleeping, related to significant daytime effects, experiencing three or more times a week for over 3 months (1). Worldwide, epidemiological research report the disease rate of insomnia to be from 10 to 12% (2–4). Although its disease rate is high, spontaneous improvement of the disorder is low. Insomnia persisted a year later in 74% of participants with insomnia and the disorder persisted for over 3 years in 46% (5). The recently revised Diagnostic and Statistical Manual of Mental Disorders, 5th edition (DSM-5) underline the need for clinical notice of sleep disorders (1). This recommendation is supported by study clarifying not only that incidences of mental and physical health co-morbidities are high, but that insomnia is an factor associated with the mood disorder (6), cardiovascular disorder (7), and hyperglycemia (8, 9). From the point of population health, to sleep is a more important issue than has been recognized so far (10, 11).

Generically, insomnia is related to worsening fatigue, work efficiency, quality of life. (12–14). In spite of these evidences of decreased function being due to sleep deficit and being a necessary diagnostic standard for insomnia, Little research has been done comparatively on its association with quality of life. Quite amazingly insomnia's perceived difficulty on personal functioning serves as an important driver of complaints and of asking for help rather than merely perceived sleep deficit (15, 16). In the epidemiological research, 20 % most generally cited reasons for visiting a sleep disorder clinic were daytime influences of fatigue, psychological suffering, physical uncomfortable feeling, and impaired work efficiency (16). The studies by the clinician of consultations furthermore show that patients with insomnia complain of disturbance of mood and cognitive abilities, coexisted with severe levels of anxiousness, fatigue, pain and physical uncomfortable feeling. Therefore, once a severe damage on quality of life (QOL) is occurred, the individuals may hope to ask for consultation (17, 18).

Pharmacotherapy for insomnia

Even though the causes of insomnia are at present inadequately understood, one of the most generally treatments provided by primary doctor is drugs including benzodiazepines, non-benzodiazepines, orexin receptor antagonists, melatonin receptor agonists, and antidepressants (19). International research shows that doctors excess dose

For peer review only - http://bmjopen.bmj.com/site/about/guidelines.xhtml

of benzodiazepine in Japan (20). Unfortunately, pharmacotherapy has a high prevalence of adverse effects. Daytime sleepiness, risk of addiction and abuse, and reoccurrence insomnia are a few examples of these side effects (21). Pharmacotherapy is for this reason not recommended (21). Recent years, two other interventions have been experimentally verified. The first intervention is cognitive behavioral therapy (CBT) for patients with insomnia. CBT has similar or even better and more continuing outcomes than pharmacological therapy, and lacks the negative side effects of pharmacological treatments (22). A second type of intervention is chronobiological treatment to enhance regular input into the body's circadian rhythms by use of bright light (23).

CBT for insomnia

CBT, regarded as the first-choice treatment for persistent sleep deficit (24, 25), is a psychological treatment aimed to correct negative thought patterns and behavior that influence to stay in insomnia. CBT is composed of a variety of methods including a behavioral part (sleep restriction therapy, stimulus control therapy, relaxation) in combination with a cognitive (managing anxiety about sleep and intrusive thoughts) and an educational (sleep hygiene education) part. Analyses of evidence of CBT for patient with insomnia show that CBT has moderate to large effects on efficiency of sleep, quality of sleep, latency to sleep onset, and time of awakening (26–28). Furthermore, a systemic review suggested that CBT for insomnia is effective compared with medication by low- to moderate-grade evidence and that about 60% of patients receiving CBT respond to therapy and 39% become remission status (29). There is a need to examine the effect of CBT on the daytime symptoms of the patients with insomnia. There is some preliminary evidence that CBT may bring generalized benefits (30–33), and even some exploratory data from small samples suggesting that CBT for patient with insomnia may reduce symptom of anxiety and depression (34, 35). However, an sufficient powered, planned trial examining sleeping condition and QOL is required. Okajima et al. showed that CBT with behavioral analysis for insomnia is more effective for insomnia and co-morbid depressive symptoms than conventional treatment and that it promotes an earlier reduction in daily doses of hypnotic medication in chronic insomnia resistant to pharmacological treatment (36).

Internet-delivered computerized cognitive behavioral therapy (ICBT) for insomnia

The recent studies use a CBT for insomnia as a internet-delivered computerized program. The choice of a web application was motivated by the observation that online programs allow a large number of individuals to access the intervention. Web programs

For peer review only - http://bmjopen.bmj.com/site/about/guidelines.xhtml

are accessible at a low cost, on one's own time. They are also preferred over generally face-to-face interventionsfor their ability to be access anonymously, convenience, and ability to be used independent of user's location. In the current trial, Van Straten et al. showed that ICBT for insomnia had statistically significant effects compared to a wait-list control group on sleep quality (Pittsburgh Sleep Quality Index; PSQI), total sleep time, and sleep efficiency (37). Further, ICBT for sleep medication-resistant insomnia was significantly more effective than the control treatment in reducing insomnia severity (Insomnia Severity Index), sleep medication, and improving sleep efficiency (38). 1600 adults will be randomized to one of two conditions in an internet-delivered computerized CBT for insomnia, which has been indicated to be effective at decreasing severity of insomnia and increasing sleep efficiency (39).

Aims

In summary, this paper describes the study protocol for a randomized controlled trial to evaluate the clinical effectiveness of ICBT adjunct to usual care (UC) for patients with insomnia who remain symptomatic after pharmacotherapy, compared with UC alone (40).

METHODS AND ANALYSIS

Study design

This study was designed as a prospective randomized open-labeled endpoint trial (PROBE) with two parallel (intervention) groups consisting of (1) a 6-week treatment regime of UC alone and (2) internet CBT combined with usual care.

Participants

Inclusion criteria for this study include going to bed between 20:00–02:00; being aged18–65 years; having a primary diagnosis of insomnia according to the DSM-5; and the insomnia being at least moderate in severity, based on a PSQI score of > 5.5 after use of sleep medication including non-benzodiazepines, benzodiazepines, melatonin receptor agonists, orexin receptor antagonists, and antidepressants

Exclusion criteria include severe symptoms of anxiety or depression. Anxiety will be assessed using the anxiety subscale of the Hospital Anxiety and Depression Scale (HADS), which contains seven items. Depression will be assessed using the total

For peer review only

For peer review only - http://bmjopen.bmj.com/site/about/guidelines.xhtml

score of the Center for Epidemiological Studies-Depression scale (CES-D). Participants with a HADS score of ≥ 10 or a CES-D score of ≥ 30 will be excluded. People with psychosis, organic mental disorder, or current high risk of suicide, substance abuse or dependence within the 12 months prior to enrolment, antisocial personality disorder, or unstable medical condition will be excluded.

Eligibility procedure for participation and diagnosis

Treatment history will be confirmed by the prescribing clinician and by chart review. All participants will be evaluated by two researchers [a psychiatrist (ES) and a therapist (DS)] who will also check diagnosis and participant eligibility. Validity of the initial diagnosis and eligibility will be discussed. Participants will be reevaluated to cover important missing information based on suggestions derived from the discussion, and final diagnosis and eligibility will be confirmed by the two researchers.

Recruitment

The planned recruitment rate is two participants per month, between March 2016 and January 2018, or until a total of 30 participants are recruited through posters and leaflets placed at medical institutions in Chiba Prefecture and through web-based and newspaper advertisements. As all participants will continue to be treated by their general practitioners, participants will be required to obtain permission from their general practitioners prior to study enrolment. This study will be conducted at the academic outpatient clinic of the Cognitive Behavioral Therapy Center, Chiba University Hospital, Japan.

Interventions

UC (conventional treatment)

There will be no restrictions on treatment options for participants who receive treatments, including medication changes, from their general practitioners. General practitioners will be permitted to refer participants for counseling or to secondary care, if deemed clinically appropriate. However, the initiation of a strictly structured internet CBT program will be prohibited in order to properly assess effectiveness of the current CBT intervention. All changes in conventional treatment, with the reasons for these changes, will be recorded throughout the study period. As UC, both UC only and ICBT plus UC groups will receive email magazines from our research office four times over a 6-week period containing information about insomnia and sleeping drugs which are generally provided by doctors. Magazine content includes: (1) “What you need to know

For peer review only - http://bmjopen.bmj.com/site/about/guidelines.xhtml

about insomnia." (week 1); (2) "What you need to know about sleeping drugs." (week 3); (3) "How to use sleeping drugs correctly" (week 5); and (4) "How to get off sleeping drugs in the near future" (week 6).These PDF magazines developed by our team will be sent by email.

ICBT program

The ICBT program for insomnia was developed by one of the authors (ES). Treatment consists of five weekly lessons and includes various elements that are commonly incorporated in common face-to-face CBT for insomnia. The central parts of this protocol include: (1) recording a sleep diary and understanding sleep hygiene; (2) changing sleep-related behaviors, including stimulus control; (3) restructuring distorted beliefs about sleep and related worries; (4) sleep restriction to increase sleep efficiency; and (5) relaxation training including breathing exercises and progressive muscle relaxation. Participants will complete the five lessons over the 6-week period to give them extra time to learn CBT.

Every lesson will contain information, examples of other people carrying out the CBT treatment, and homework. One of the researchers (DS) is a cognitive behavioral therapist and will send weekly emails to participants to ask them about their progress. The aim of this e-mail support by a therapist is to allow participants to comment on the ICBT exercise, clarify information, and motivate the participant to persist in carrying out ICBT and the requested homework. Participants will also be permitted to send emails to DS if they have questions about the content provided.

Outcomes

Baseline and clinical characteristics

Baseline characteristics will include gender, age, education, marital status, employment status, age at insomnia onset, and duration of insomnia. Moreover, treatment history will include the names of medications to which the participant has exhibited resistance, other prior treatments, current treatment (medication and others) at baseline and all changes in conventional treatment throughout the study period.

Primary outcome

The primary outcome will be PSQI score at week 6. PSQI is a self-rated questionnaire with 19 questions, and consists of seven subscales (sleep quality, sleep latency, sleep duration, habitual sleep efficiency, sleep disturbance, use of sleep medication, and daytime dysfunction). Each subscale is scored on a scale of 0 to 3.

For peer review only - http://bmjopen.bmj.com/site/about/guidelines.xhtml

Subscale scores are summed to a total score ranging from 0 (good quality of sleep) to 21 (very poor quality of sleep). PSQI was verified as a reliable and valid measure subjective sleep quality in clinical practice and research (41, 42).

Secondary outcomes

Secondary outcomes will be: sleep onset latency, total sleep time, and sleep efficiency extracted from PSQI, as well as current feeling of refreshment and soundness of sleep measured by visual analog scale, number of awakenings as measured by HADS as a measure of anxiety, CES-D to measure depression, and Euro Qol-5D (EQ-5D) as a measure of QOL.

The anxiety score of the seven anxiety items from HADS range from 0 (no symptoms of anxiety) to 21 (severe symptoms of anxiety). The total score of the 20 CES-D items ranges from 0 (no symptoms of depression) to 60 (severe symptoms of depression).The 3-level version of EQ-5D is described in our previous study (Seki et al.) (43). The EQ-5D index (43) contains five items that assess QOL on a 3-point Likert scale ranging from 1 (not severe) to 3 (severe). The Japanese version of EQ-5D was developed by Tsuchiya et al. (44). EQ-5D is the most commonly used scale internationally for calculating quality-adjusted life years (QALYs). QALYs are often used in cost-utility analyses as the health outcome of choice; they are typically estimated via area under the curve analysis, which involves summing the areas of the distribution shapes for utility scores over the study period (43, 45). In the present study, QALYs will be assessed using EQ-5D, an indicator of patient health status. This index is calculated by transforming the EQ-5D dimension scores into a single summary score ranging from 0 to 1 (1 = full health) by applying a formula created by the EuroQol Group (43, 46). Participants will complete these questionnaires at home.

The therapist will ask participants about adverse event experiences at each assessment.

All measures will be assessed at weeks 0 (baseline), 6 (post-intervention), and 12 (follow-up) and outcomes will be analyzed based on the intention-to-treat principle.

Sample size

This sample size was based on a previous study reported by van Straten et al. (38) which indicated that the estimated group difference in changes of PSQI scores from baseline was approximately 2.86 (ICBT group = 3.00 vs. control group = 0.04). The conventional treatment (i.e., UC) alone is assumed to be largely ineffective. Assuming a

For peer review only - http://bmjopen.bmj.com/site/about/guidelines.xhtml

group difference of 2.86 points [standard deviation (SD) = 2.5], 13 subjects per group will provide 80% power to detect a difference in PSQI scores between the UC arm and the arm with internet CBT combined with UC for at least 6 weeks, using a two-sided, two-sample t-test at a 5% significance level. Thus, allowing for a 10% dropout rate, 15 participants are required per group, for a total of 30 participants in the study.

Randomization

At the end of the baseline assessment, eligible participants will be randomly assigned to either the UC arm or ICBT plus US arm at a ratio of 1:1, with assignments made using the minimization method, ensuring a balance in baseline PSQI scores (PSQI ≥ 12) and gender.

Each participant will then be assigned to one of the two treatment regimes. Participants will be blinded to the group to which they are assigned before they consent to participate in the study.

Data analysis plan

Statistical analysis and reporting of this trial will be conducted in accordance with CONSORT guidelines, with primary analyses based on the intention-to-treat principle. For baseline variables, summary statistics will be constructed, employing frequencies and proportions for categorical data, and mean and SD for continuous variables. Baseline variables will be compared using Fisher's exact test for categorical outcomes and unpaired t-test for continuous variables.

For the primary analysis comparing the treatment effects, the least squares means (LSMean) and their 95% confidence interval (CI), which will be estimated by analysis of covariance (ANCOVA) with the change in the PSQI total scores at week 6. This ANCOVA model will be carried out taking into account the variation caused by treatment effects, and using sex, and baseline PSQI score as covariates. Analyses of secondary outcomes will be performed in the same manner as the primary analysis.

All comparisons are planned, and all p-values are two-sided. A p-value < 0.05 is considered statistically significant. All statistical analyses are performed using the SAS software program, version 9.4 (SAS Institute, Cary, NC, USA).

Ethics and dissemination

For peer review only - http://bmjopen.bmj.com/site/about/guidelines.xhtml

This study will be conducted at the Academic Outpatient Clinic of the Cognitive Behavioral Therapy Center, Chiba University Hospital, Japan. The study protocol has been approved by the Institutional Review Board of the Chiba University Hospital (reference number: G27040) on December, 2015.

When potential participants contact the study trial office, they will be informed of the study objectives and asked if they are willing to participate. Each patient will be informed that participation is voluntary and that full anonymity will be provided. Each participant will then be required to provide written informed consent for their participation in this study. Each participant will also be informed that all participants will receive UC (conventional treatment) from their general practitioner; half of the recruited individuals will also receive ICBT in addition to their UC. A doctor's examination will be conducted at each assessment point (weeks 0, 6, and 12).

An adverse event could consist of any unfavorable and unintended sign, symptom, or disease temporally associated with this interventional study, whether or not considered related to this ICBT, and all adverse events will be reported. Serious adverse events will be immediately reported to the Institutional Review Board of Chiba University Hospital in addition to be registered with the hospital risk management system. Moreover, an independent data monitoring committee will accurately verify detailed record of the progress of the clinical study, critical efficacy variables, and safty data, and recommend the sponsor to continue, modify, or terminate the study.

Study outcomes will be published in international journals, regardless of the outcome. The study will be carried out and reported according to the CONSORT recommendations.

DISCUSSION

This pilot study was planned to address the lack of trial on the adoption of ICBT for insomnia after hypnotic treatment. Moreover, findings of this study will provide valuable evidence to facilitate the development of second-line treatments after pharmacotherapy and to establish treatment algorithms. The limitations of this study are the following. First, sleep estimates will be based on subjective sleep diaries and PSQI, rather than on objective measures such as polysomnography or actigraphy. The use of both subjective and objective measures has been recommended because people with

For peer review only

insomnia underestimate their actual sleep time (11, 17). However, using polysomnography is costly and imposes burden on participants. Therefore, sleep diaries are currently the most widely used outcome measure in insomnia treatment studies (5) and are generally well accepted because subjective complaints generally prompt participants to seek treatment. The second limitation is that we will be unable to elucidate specific effects of the ICBT program because a psychological placebo group to control for nonspecific factors will not be employed in this study.

For peer review only

For peer review only - http://bmjopen.bmj.com/site/about/guidelines.xhtml

References

1. American Psychiatric Association. Diagnostic and statistical manual of mental disorders. 5th ed. Washington: American Psychiatric Association; 2013.
2. Ohayon MM. Epidemiology of insomnia: what we know and what we still need to learn. Sleep Med Rev. 2002; 6: 97-111.
3. Lichstein KL, Durrence HH, Reidel BW, et al. The epidemiology of sleep: age, gender and ethnicity. Mahwah: Lawrence Erlbaum Associates; 2004.
4. Morphy H, Dunn KM, Lewis M, et al. Epidemiology of insomnia: a longitudinal study in a UK Population. Sleep. 2007; 30 (3): 274–80.
5. Morin CM, Belanger L, LeBlanc M, et al. The natural history of insomnia: a population-based 3-year longitudinal study. Arch Intern Med. 2009; 169: 447-53.
6. Baglioni C, Battagliese G, Feige B, et al. Insomnia as a predictor of depression: a meta-analytic evaluation of longitudinal epidemiological studies. J Affect Disord. 2011; 135: 10-9.
7. Vgontzas AN, Liao D, Bixler EO, Chrousos GP, Vela-Bueno A. Insomnia with objective short sleep duration is associated with a high risk for hypertension. Sleep. 2009; 32: 491-7.
8. Vgontzas AN, Liao D, Pejovic S, Calhoun S, Karataraki M, Bixler EO. Insomnia with objective short sleep duration is associated with type 2 diabetes: a population-based study. Diabetes Care. 2009; 32: 1980-5.
9. Cappuccio FP, D'Elia L, Strazzullo P, Miller MA. Quantity and quality of sleep and incidence of type 2 diabetes. A systematic review and meta-analysis. Diabetes Care. 2010; 33 (2): 414-20.
10. Luyster FS, Strollo PJ, Zee PC, on behalf of the Boards of Directors of the American Academy of Sleep Medicine and the Sleep Research Society JK. Sleep: a health imperative. Sleep. 2012; 35 (6): 727-34.
11. Buysse DJ. Sleep health: can we define it? Does it matter? Sleep. 2014; 37 (1): 9-17.
12. Roth T, Ancoli-Israel S. Daytime consequences of insomnia in the United States: results of the 1991 National Sleep Foundation Survey II. Sleep. 1999; 22 Suppl 2: S354-63.
13. Kyle SD, Morgan K, Espie C. Insomnia and health-related quality of life. Sleep Med Rev. 2010; 14: 69-82.
14. Espie CA, Kyle SD, Hames P, et al. The daytime impact of DSM-5 insomnia disorder: comparative analysis of insomnia subtypes from the Great British Sleep Survey. J Clin Psychiatry. 2012; 73: e1478-84.

For peer review only - http://bmjopen.bmj.com/site/about/guidelines.xhtml

15. Stepanski E, Koshorek G, Zorick F, Glinn M, Roehrs T, Roth T. Characteristics of individuals who do or do not seek treatment for chronic insomnia. Psychosomatics. 1989; 30: 421-7.
16. Morin CM, LeBlanc M, Daley M, Gregoire JP, Mérette C. Epidemiology of insomnia: prevalence, self-help treatments, consultations, and determinants of help-seeking behaviors. Sleep Med 2007; 7 (2): 123-30.
17. Buysse DJ, Thompson W, Scott J, Franzen PL, Germain A, Hall M, et al. Daytime symptoms in primary insomnia: a prospective analysis using ecological momentary assessment. Sleep Med 2007; 8 (3): 198-208.
18. Levitt H, Wood A, Moul DE, Hall M, Germain A, Kupfer DJ, et al. A pilot study of subjective daytime alertness and mood in primary insomnia participants using ecological momentary assessment. Behav Sleep Med 2004; 2 (2): 113-31.
19. Morin CM, Benca RM. Chronic insomnia. Lancet. 2012; 379: 1129-41.
20. International Narcotics Control Board. Report of the International Narcotics Control Board on the Availability of Internationally Controlled Drugs: Ensuring Adequate Access for Medical and Scientific Purposes. 2010; 32-41. http://apps.who.int/medicinedocs/en/m/abstract/Js21969en/.
21. Benca RM. Diagnosis and Treatment of Chronic Insomnia: A Review. Psychiatr Serv. 2005; 56: 332-43.
22. National Institutes of Health. National Institutes of Health State of the Science Conference Statement on manifestations and management of chronic insomnia in adults 2005. Sleep 2005; 28: 1049-57.
23. der Lek RF R-v, Swaab DF, Twisk J, Hol EM, Hoogendijk WJG, Someren EJWV. Effect of bright light and melatonin on cognitive and noncognitive function in elderly residents of group care facilities. JAMA 2008;299:2642–55.
24. Morin CM, Vallières A, Guay B, Ivers H, Savard J, Mérette C, et al. Cognitive behavioral therapy, singly and combined with medication, for persistent insomnia. JAMA 2009; 301: 2005-15.
25. NIH. State-of-the-Science Conference Statement on Manifestations and Management of Chronic Insomnia in Adults. NIH Consens Sci Statements 2005; 22: 1-30.
26. Wilson SJ, Nutt DJ, Alford C, et al. British Association for Psychopharmacology consensus statement on evidence-based treatment of insomnia, parasomnias and circadian rhythm disorders. J Psychopharmacol 2010; 24: 1577-600.

For peer review only

For peer review only - http://bmjopen.bmj.com/site/about/guidelines.xhtml

27. Trauer JM, Qian MY, Doyle JS, Rajaratnam SMW, Cunnington D. Cognitive behavioral therapy for chronic insomnia: a systematic review and metaanalysis. Ann Intern Med. 2015; 163: 191-204.
28. Wu JQ, Appleman ER, Salazar RD, Ong JC. Cognitive behavioral therapy for insomnia comorbid with psychiatric and medical conditions: a metaanalysis. JAMA Intern Med 2015; 175: 1461-72.
29. Mitchell MD, Gehrman P, Perlis M, Umscheid CA. Comparative effectiveness of cognitive behavioral therapy for insomnia: a systematic review. BMC Fam Pract 2012; 13 (1): 40.
30. Morin CM, Vallières A, Guay B, Ivers H, Savard J, Mérette C, et al. Cognitivebehavior therapy, singly and combined with medication, for persistent insomnia: acute and maintenance therapeutic effects. JAMA 2009; 301 (19): 2005-15.
31. Espie CA, Inglis SJ, Tessier S, Harvey L. The clinical effectiveness of cognitive behaviour therapy for chronic insomnia: implementation and evaluation of a sleep clinic in general medical practice. Behav Res Ther. 2001; 39 (1) :45-60.
32. Espie CA, MacMahon KM, Kelly H, et al. Randomized clinical effectiveness trial of nurse-administered small group cognitive behaviour therapy for persistent insomnia in general practice. Sleep 2007; 30: 574-84.
33. Espie CA, Fleming L, Cassidy J, et al. Randomized controlled clinical effectiveness trial of cognitive behaviour therapy compared with treatment as usual for persistent insomnia in patients with cancer. J Clin Oncol 2008; 26: 4651-8.
34. Espie CA, Kyle SD, Miller CB, Ong J, Hames P, Fleming L. Attribution,cognition and psychopathology in persistent insomnia disorder: outcome and mediation analysis from a randomized placebo-controlled trial of online cognitive behavioural therapy. Sleep Med 2014; 15 (8): 913-7.
35. Manber R, Edinger JD, Gress JL, San Pedro-Salcedo MG, Kuo TF, Kalista T. Cognitive behavioral therapy for insomnia enhances depression outcome in patients with comorbid major depressive disorder and insomnia. Sleep 2008; 31: 489-95.
36. Okajima, I., Nakamura, M., Nishida, S., Usui, A., Kanno, M., Nakajima, S., & Inoue, Y. Cognitive behavioural therapy with behavioural analysis for pharmacological treatment-resistant chronic insomnia. Psychiatry Research 2013; 210(2): 515-521.
37. Ritterband LM, Thorndike FP, Gonder-Frederick LA. et al. Efficacy of an Internet-based behavioral intervention for adults with insomnia. Arch Gen Psychiatry 2009; 66(7): 692–698.

For peer review only - http://bmjopen.bmj.com/site/about/guidelines.xhtml

38. Van Straten, Emmelkamp, de Wit, Lancee, Andersson, van Someren EJ, Cuijpers P. Guided Internet-delivered cognitive behavioural treatment for insomnia: a randomized trial. Psychol Med 2014; 44(7): 1521-32.
39. Kaldo V, Jernelöv S, Blom K, Ljótsson B, Brodin M, Jörgensen M, Kraepelien M, Rück C, Lindefors N. Guided internet cognitive behavioral therapy for insomnia compared to a control treatment - A randomized trial. Behav Res Ther 2015; 71: 90-100.
40. Yoshinaga N, Niitsu T, Hanaoka H, Sato Y, Ohshima F, Matsuki S, Kobori O, Nakazato M, Nakagawa A, Iyo M, Shimizu E. Strategy for treating selective serotonin reuptake inhibitor-resistant social anxiety disorder in the clinical setting: a randomised controlled trial protocol of cognitive behavioural therapy in combination with conventional treatment. BMJ Open. 2013 Feb 13; 3(2): pii: e002242.
41. Carpentera J, Andrykowskia M. Psychometric evaluation of the pittsburgh sleep quality index. Journal of Psychosomatic Research 1998; 45(1): 5-13.
42. Doi Y, Minowa M, Uchiyama M, Okawa M, Kim K, Shibui K, Kamei Y. Psychometric assessment of subjective sleep quality using the Japanese version of the Pittsburgh Sleep Quality Index (PSQI-J) in psychiatric disordered and control subjects. Psychiatry Research 2000; 97(2-3): 165-72.
43. Seki Y, Nagata S, Shibuya T, Yoshinaga N, Yokoo M, Ibuki H, Minamitani N, Kusunoki M, Inada Y, Kawasoe N, Adachi S, Yoshimura Y, Nakazato M, Iyo M, Nakagawa A, Shimizu E. A Feasibility Study of the Clinical Effectiveness and Cost-Effectiveness of Individual Cognitive Behavioral Therapy for Panic Disorder in a Japanese Clinical Setting: An Uncontrolled Pilot Study. BMC Research Notes 2016; 9(1): 458.
44. Tsuchiya A, Ikeda S, Ikegami N, Nishimura S, Sakai I, Fukuda T, Hamashima C, Hisashige A, Tamura M. Estimating an EQ-5D population value set: the case of Japan. Health Econ 2002; 11: 341–53.
45. EuroQol Group. A new facility for the measurement of health-related quality of life. Health Policy. 1990; 16: 199–208.
46. Manca A, Hawkins N, Sculpher MJ. Estimating mean QALYs in a trial-based cost-effectiveness analysis: the importance of controlling for baseline utility. Health Econ 2005; 14: 487–96.

Authors' affiliations

[1]Departments of Cognitive Behavioral Physiology Graduate School of Medicine, Chiba University, Chiba, Japan

For peer review only - http://bmjopen.bmj.com/site/about/guidelines.xhtml

[2]Department of Rehabilitation Sciences, Faculty of Health Care Sciences, Chiba Prefectural University of Health Sciences, Chiba, Japan
[3]Organization for Promotion of Tenure Track, University of Miyazaki, Miyazaki, Japan
[4]Clinical Research Center, Chiba University Hospital, Chiba, Japan
[5]Department of Global Clinical Research / Biostatistics, Graduate School of Medicine, Chiba University, Chiba, Japan
[6]Research Center for Child Mental Development, Chiba University, Chiba, Japan
[7]Cognitive Behavioral Therapy Center, Chiba University Hospital, Chiba, Japan

Contributors Statement

Daisuke Sato and Eiji Shimizu designed the study, and wrote the initial draft of the manuscript. Yasunori Sato contributed to analysis and interpretation of data, and assisted in the preparation of the manuscript. All other authors have contributed to data collection and interpretation, and critically reviewed the manuscript. All authors approved the final version of the manuscript, and agree to be accountable for all aspects of the work in ensuring that questions related to the accuracy or integrity of any part of the work are appropriately investigated and resolved.

Funding statement None.

Competing interests statement None.

For peer review only

For peer review only - http://bmjopen.bmj.com/site/about/guidelines.xhtml

Figure 1 Patient flow during inclusion, randomization and treatment.

CONSORT Flow Diagram

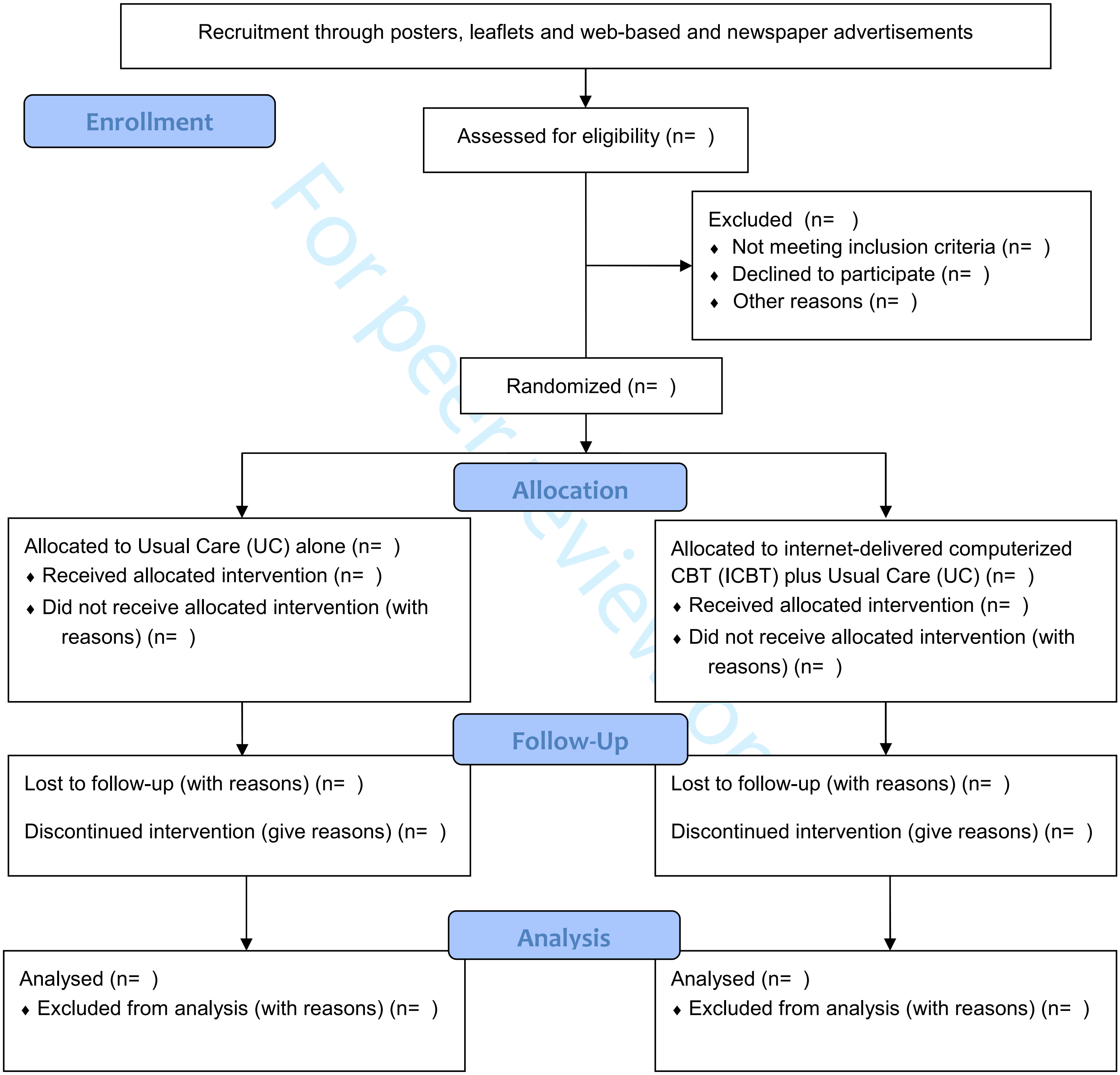

For peer review only

CONSORT 2010 checklist of information to include when reporting a randomised trial*

Section/Topic	Item No	Checklist item	Reported on page No
**Title and abstract**			
	1a	Identification as a randomised trial in the title	P1
	1b	Structured summary of trial design, methods, results, and conclusions (for specific guidance see CONSORT for abstracts)	P2
**Introduction**			
Background and objectives	2a	Scientific background and explanation of rationale	P4 – 6
	2b	Specific objectives or hypotheses	P6
**Methods**			
Trial design	3a	Description of trial design (such as parallel, factorial) including allocation ratio	P6
	3b	Important changes to methods after trial commencement (such as eligibility criteria), with reasons	None
Participants	4a	Eligibility criteria for participants	P6 – 7
	4b	Settings and locations where the data were collected	P7
Interventions	5	The interventions for each group with sufficient details to allow replication, including how and when they were actually administered	P7 – 8
Outcomes	6a	Completely defined pre-specified primary and secondary outcome measures, including how and when they were assessed	P8 – 9
	6b	Any changes to trial outcomes after the trial commenced, with reasons	None
Sample size	7a	How sample size was determined	P9 – 10
	7b	When applicable, explanation of any interim analyses and stopping guidelines	Non-applicable

For peer review only - http://bmjopen.bmj.com/site/about/guidelines.xhtml

Randomisation:			P10
Sequence generation	8a	Method used to generate the random allocation sequence	P10
	8b	Type of randomisation; details of any restriction (such as blocking and block size)	P10
Allocation concealment mechanism	9	Mechanism used to implement the random allocation sequence (such as sequentially numbered containers), describing any steps taken to conceal the sequence until interventions were assigned	P10
Implementation	10	Who generated the random allocation sequence, who enrolled participants, and who assigned participants to interventions	P10
Blinding	11a	If done, who was blinded after assignment to interventions (for example, participants, care providers, those assessing outcomes) and how	P10
	11b	If relevant, description of the similarity of interventions	non-applicable
Statistical methods	12a	Statistical methods used to compare groups for primary and secondary outcomes	P10
	12b	Methods for additional analyses, such as subgroup analyses and adjusted analyses	P10
**Results**			
Participant flow (a diagram is strongly recommended)	13a	For each group, the numbers of participants who were randomly assigned, received intended treatment, and were analysed for the primary outcome	None
	13b	For each group, losses and exclusions after randomisation, together with reasons	None
Recruitment	14a	Dates defining the periods of recruitment and follow-up	None
	14b	Why the trial ended or was stopped	None
Baseline data	15	A table showing baseline demographic and clinical characteristics for each group	None
Numbers analysed	16	For each group, number of participants (denominator) included in each analysis and whether the analysis was by original assigned groups	None
Outcomes and estimation	17a	For each primary and secondary outcome, results for each group, and the estimated effect size and its precision (such as 95% confidence interval)	None
	17b	For binary outcomes, presentation of both absolute and relative effect sizes is recommended	None
Ancillary analyses	18	Results of any other analyses performed, including subgroup analyses and adjusted analyses, distinguishing pre-specified from exploratory	None
Harms	19	All important harms or unintended effects in each group (for specific guidance see CONSORT for harms)	None
**Discussion**			
Limitations	20	Trial limitations, addressing sources of potential bias, imprecision, and, if relevant, multiplicity of analyses	P11
Generalisability	21	Generalisability (external validity, applicability) of the trial findings	None
Interpretation	22	Interpretation consistent with results, balancing benefits and harms, and considering other relevant evidence	None

For peer review only - http://bmjopen.bmj.com/site/about/guidelines.xhtml

**Other information**			
Registration	23	Registration number and name of trial registry	P2
Protocol	24	Where the full trial protocol can be accessed, if available	P2 Please refer to the following URL https://upload.umin.ac.jp/cgi-open-bin/ctr/ctr_view.cgi?recptno=R000023545
Funding	25	Sources of funding and other support (such as supply of drugs), role of funders	P17

*We strongly recommend reading this statement in conjunction with the CONSORT 2010 Explanation and Elaboration for important clarifications on all the items. If relevant, we also recommend reading CONSORT extensions for cluster randomised trials, non-inferiority and equivalence trials, non-pharmacological treatments, herbal interventions, and pragmatic trials. Additional extensions are forthcoming: for those and for up to date references relevant to this checklist, see www.consort-statement.org.

For peer review only

For peer review only - http://bmjopen.bmj.com/site/about/guidelines.xhtml

BMJ Open

Study protocol for a pilot randomized controlled trial on the effect of internet-delivered computerized cognitive behavioral therapy on patients with insomnia who remain symptomatic following hypnotics

Journal:	*BMJ Open*
Manuscript ID	bmjopen-2017-018220.R1
Article Type:	Protocol
Date Submitted by the Author:	23-Oct-2017
Complete List of Authors:	Sato, Daisuke; Chiba University Graduate School of Medicine, Departments of Cognitive Behavioral Physiology; Chiba Prefectural University of Health Sciences, Rehabilitation Sciences<br>Yoshinaga, Naoki; Miyazaki Daigaku, Organization for Promotion of Tenure Track<br>Nagai, Eichi; Chiba University Hospital, Clinical Research Center<br>Hanaoka, Hideki; Chiba University Hospital, Clinical Research Center<br>Sato, Yasunori; Chiba University Graduate School of Medicine, Department of Global Clinical Research / Biostatistics<br>Shimizu, Eiji; Chiba University, Department of Cognitive Behavioral Physiology, Graduate School of Medicine; Chiba University, Research Center for Child Mental Development
**Primary Subject Heading**:	Mental health
Secondary Subject Heading:	Evidence based practice, Patient-centred medicine, Mental health
Keywords:	Cognitive Behavioral Therapy, Insomnia, Randomized Controlled Trial, Internet

SCHOLARONE™
Manuscripts

Study protocol for a pilot randomized controlled trial on the effect of internet-delivered computerized cognitive behavioral therapy on patients with insomnia who remain symptomatic following hypnotics

Daisuke Sato,[1, 2] Naoki Yoshinaga,[3] Eiichi Nagai,[4] Hideki Hanaoka,[4] Yasunori Sato,[5] Eiji Shimizu[1, 6, 7]

1) Departments of Cognitive Behavioral Physiology, Chiba University Graduate School of Medicine, Chiba, Japan
2) Department of Rehabilitation Sciences, Faculty of Health Care Sciences, Chiba Prefectural University of Health Sciences, Chiba, Japan
3) Organization for Promotion of Tenure Track, University of Miyazaki, Miyazaki, Japan
4) Clinical Research Center, Chiba University Hospital, Chiba, Japan
5) Department of Global Clinical Research / Biostatistics, Graduate School of Medicine, Chiba University, Chiba, Japan
6) Research Center for Child Mental Development, Chiba University, Chiba, Japan
7) Cognitive Behavioral Therapy Center, Chiba University Hospital, Chiba, Japan

Corresponding author

Daisuke Sato,[1, 2]

1) Departments of Cognitive Behavioral Physiology, Chiba University Graduate School of Medicine, Chiba, Japan
2) Department of Rehabilitation Sciences, Faculty of Health Care Sciences, Chiba Prefectural University of Health Sciences, Chiba, Japan

Departments of Cognitive Behavioral Physiology, Chiba University Graduate School of Medicine, Chiba, Japan

1-8-1 Inohana, Chuo-ku, Chiba 260-8670, Japan

Tel: (+81) 43-226-2027, Fax: (+81) 43-226-2028

E-mail: daisu.sato@gmail.com

Keywords: Cognitive Behavioral Therapy, Insomnia, Randomized Controlled Trial, Internet

For peer review only - http://bmjopen.bmj.com/site/about/guidelines.xhtml

Word count: 3595

Number of tables and figures: 1

Protocol version: ver.4.0 (20-Oct-2016)

For peer review only

For peer review only - http://bmjopen.bmj.com/site/about/guidelines.xhtml

ABSTRACT

Introduction: Insomnia has severe consequences for health. Primary care physicians in Japan commonly provide hypnotics, which is far from optimal. The recommended treatment for insomnia is cognitive behavioral therapy (CBT). Access to trained therapists, however, is limited. Rather than face-to-face CBT, several researchers have studied internet-delivered computerized CBT (ICBT). This paper describes the study protocol for a pilot randomized controlled trial (RCT) to evaluate effectiveness and feasibility of our newly developed five-step ICBT as an adjunct to usual care (UC) compared with UC alone for patients with insomnia who remain symptomatic following hypnotics.

Methods and analysis: This proposed exploratory RCT comprises two parallel groups (ICBT + UC and UC alone) consisting of 15 participants each (n = 30) diagnosed with insomnia who remain symptomatic after pharmacotherapy. We aim to evaluate the effectiveness of 6 intervention weeks. The primary outcome of insomnia severity will be the Pittsburgh Sleep Quality Index (PSQI) at week 6. Secondary outcomes include sleep onset latency, total sleep time, sleep efficiency extracted from PSQI, current feeling of refreshment and perceived soundness of sleep measured using visual analog

For peer review only - http://bmjopen.bmj.com/site/about/guidelines.xhtml

scale, number of awakenings, anxiety by Hospital Anxiety and Depression Scale, depression by Center for Epidemiologic Studies Depression Scale, and quality of life by Euro Qol-5D. All measures will be assessed at weeks 0 (baseline), 6 (post-intervention), and 12 (follow-up), and intention-to-treat will be applied. The statistical analysis plan has been developed considering design of field materials.

Ethics and dissemination: This study will be conducted at the academic outpatient clinic of Chiba University Hospital, Japan. Ethics approval was granted by the Institutional Review Board of Chiba University Hospital. All participants will be required to provide written informed consent. The trial will be implemented and reported in accordance with CONSORT recommendations.

Clinical Trial Registration Number: UMIN000021509

For peer review only

ARTICLE SUMMARY

Strengths and limitations of this study

- This is the first pilot RCT to focus on ICBT as a therapeutic option for insomnia patients who remain symptomatic following hypnotics.
- This pilot study reflects good clinical practice, and its results will contribute to the development of second-line treatments and establish future treatment algorithms.
- A study limitation is the inability to elucidate specific effects of the ICBT program because a psychological placebo group to control for nonspecific factors will not be employed and sleep estimates will not be based on objective measures such as polysomnography or actigraphy.

For peer review only

INTRODUCTION

Insomnia is characterized by the inability to fall asleep or awakening too early in the morning or during the night, resulting in non-restorative sleep and decreased daytime functioning. Insomnia comprises a symptomatic state of difficulty in sleeping, related to significant daytime effects, experienced three or more times a week for over 3 months (1). Worldwide epidemiological research reports the incidence of insomnia to be 10%–12% (2–4). Although the incidence of insomnia is high, spontaneous improvement of the disorder is low. In one study, insomnia persisted a year later in 74% of participants with insomnia and the disorder persisted for over 3 years in 46% (5). The recently revised Diagnostic and Statistical Manual of Mental Disorders, 5th edition (DSM-5) underlines the need for clinical acknowledgment of sleep disorders (1). This recommendation is supported by studies reporting not only that the incidence of mental and physical health co-morbidities are high but that insomnia is a factor associated with mood disorders (6), cardiovascular disease (7), and hyperglycemia (8, 9). From a population health perspective, sleep is a more important issue than was previously recognized (10, 11).

For peer review only - http://bmjopen.bmj.com/site/about/guidelines.xhtml

In general, insomnia is related to worsening fatigue, work efficiency, and decreased quality of life (QOL) (12–14). In spite of this evidence of decreased productivity resulting from sleep deficits and it being a necessary diagnostic standard for insomnia, comparatively little research has been conducted on its association with QOL. Quite amazingly while insomnia's perceived sleep deficit is few, many people begin asking for help, particularly when insomnia's perceived difficulty on personal functioning such as daytime fatigue become more pronounced. (15, 16). In epidemiological research, 20% of the most frequently cited reasons for visiting a sleep disorder clinic were daytime symptoms of fatigue, psychological suffering, physical discomfort as heartburn, a backflow of acid and food from the stomach into the esophagus after eating, and impaired work efficiency (16). Moreover, studies performed by sleep clinicians at consultations show that patients with insomnia complain of disturbances of mood and cognitive abilities coexisting with severe levels of anxiousness, fatigue, pain, and physical discomfort. Once QOL has been severely impacted, individuals often request a consultation (17, 18).

Pharmacotherapy for insomnia

For peer review only - http://bmjopen.bmj.com/site/about/guidelines.xhtml

Although the causes of insomnia are at present inadequately understood, some of the most common treatments provided by primary physicians are drugs including benzodiazepines, non-benzodiazepines, orexin receptor antagonists, melatonin receptor agonists, and antidepressants (19). Furthermore, international research shows that physicians in Japan prescribe excess doses of benzodiazepine (20). Unfortunately, pharmacotherapy is associated with a high incidence of adverse effects, and examples of side effects include daytime sleepiness and recurrence insomnia (21). For this reason, pharmacotherapy is not recommended (21). In recent years, two alternate types of intervention for patients with insomnia have been experimentally examined. The first is cognitive behavioral therapy (CBT). CBT has similar or even better and more long-lasting outcomes than pharmacological therapy and lacks the negative side effects of pharmacological treatments (22). Clinical practice guidelines suggest CBT, rather than pharmacotherapy, as the initial therapy for patients with insomnia (23–25).The second type of intervention is chronobiological treatment, which enhances regular input into the body's circadian rhythms using bright light (26). It has been reported that compared with CBT, the effect of light therapy on sleep maintenance is lower (27). Vallières A et al. reported that pharmacotherapy prior to the initiation of CBT appears to be less effective than the combined treatment of pharmacotherapy and CBT, followed

For peer review only
For peer review only - http://bmjopen.bmj.com/site/about/guidelines.xhtml

by CBT alone (28). This study also found that early introduction of CBT contributes to maximizing the effect of pharmacotherapy.

CBT for insomnia

CBT, regarded as the first-choice treatment for persistent sleep deficits (29, 2430), is a psychological treatment aiming to correct negative thought patterns and behaviors that influence the persistence of insomnia. CBT is composed of a variety of methods including a behavioral component (sleep restriction therapy, stimulus control therapy, and relaxation) in combination with cognitive (managing anxiety about sleep and intrusive thoughts) and educational (sleep hygiene education) components. Sleep restriction therapy is an approach designed to reduce the time in bed to the actual time of sleep being achieved (31). Stimulus control treatment is behavioral directions to associate the bed with sleep and to restore a consistent sleep rhythm (32). Sleep hygiene education includes health practices such as physical exercise and environmental correlates such as light that may further or disturb sleep (33). Studies examining the effect of CBT in patients with insomnia show that CBT has moderate-to-large effects on sleep efficiency and quality, latency to sleep onset, and time of awakening (34–36). Furthermore, a systemic review concluded that there is low- to moderate-grade evidence

For peer review only - http://bmjopen.bmj.com/site/about/guidelines.xhtml

suggesting that CBT for insomnia is more effective than medication and that approximately 60% of patients receiving CBT respond to therapy and 39% enter remission (37). Further research examining the effect of CBT on the daytime symptoms of patients with insomnia is needed. There is some preliminary evidence that CBT may provide generalized benefits (38–40), as well as some exploratory data from small samples suggesting that CBT for patients with insomnia may reduce anxiety and depression symptoms (41, 42). However, a sufficiently powered, well-planned trial examining the relationship between characteristics of sleep, such as sleep quality and QOL is required. Okajima et al. showed that CBT with behavioral analysis is more effective for insomnia and co-morbid depressive symptoms than conventional treatment and that it promotes an earlier reduction in daily doses of hypnotic medication in patients with chronic insomnia who are resistant to pharmacological treatment (43).

Internet-delivered computerized CBT (ICBT) for insomnia

Recent studies have administered CBT for insomnia as an internet-delivered computerized program (44). The choice of a web application is generally motivated by the observation that online programs are more accessible, have low cost, and can be completed at one's own time. They are also generally preferred over face-to-face

For peer review only - http://bmjopen.bmj.com/site/about/guidelines.xhtml

interventions because they can be accessed anonymously, are convenient, and can be used anywhere. In a recent trial, Van Straten et al. showed statistically significant effects on sleep quality (Pittsburgh Sleep Quality Index; PSQI), total sleep time, and sleep efficiency in participants receiving ICBT for insomnia compared with a wait-list control group (45). Furthermore, ICBT for hypnotics-resistant insomnia was significantly more effective than the control treatment in reducing insomnia severity (Insomnia Severity Index), use of hypnotics, and improving sleep efficiency (46).

Aims

In summary, this paper describes the study protocol for a randomized controlled trial designed to evaluate the clinical effectiveness of ICBT adjunct to usual care (UC) for patients with insomnia who remain symptomatic after pharmacotherapy, compared with UC alone (47).

METHODS AND ANALYSIS

Study design

For peer review only - http://bmjopen.bmj.com/site/about/guidelines.xhtml

This study was designed as a prospective randomized open-labeled endpoint trial (“PROBE”) with two parallel intervention groups consisting of (1) a 6-week treatment regime of UC alone and (2) an ICBT combined with UC. (Figure 1)

Participants

Inclusion criteria for this study include going to bed between 20:00 and 02:00; age, 18–65 years; having a primary diagnosis of insomnia according to the DSM-5; and the aforementioned insomnia remaining symptomatic. “Remaining symptomatic” is defined herein as having insomnia that is at least moderate in severity, based on a PSQI score of >5.5, after the use of hypnotics including non-benzodiazepines, benzodiazepines, melatonin receptor agonists, orexin receptor antagonists, and antidepressants.

Exclusion criteria include severe symptoms of anxiety or depression. Anxiety will be assessed using the anxiety subscale of the Hospital Anxiety and Depression Scale (HADS), which contains seven items. Depression will be assessed using the total score of the Center for Epidemiological Studies Depression scale (CES-D). Participants with a HADS score of ≥10 or a CES-D score of ≥30 will be excluded. Participants with

For peer review only - http://bmjopen.bmj.com/site/about/guidelines.xhtml

psychosis, organic mental disorder, or current high risk of suicide, substance abuse or dependence within the 12 months prior to enrolment, antisocial personality disorder, or unstable medical condition will also be excluded.

Eligibility procedure for participation and diagnosis

Treatment history will be confirmed by the prescribing clinician and by chart review. All participants will be evaluated by two researchers [a psychiatrist (ES) and a therapist (DS)] who will also verify patient diagnosis and eligibility. Validity of the initial diagnosis and eligibility will be discussed. Participants will be reevaluated to cover important missing information based on suggestions derived from the discussion, and final diagnosis and eligibility will be confirmed by the two researchers.

Recruitment

The planned recruitment rate is two participants per month, between March 2016 and January 2018, or until a total of 30 participants are recruited through posters and leaflets placed at medical institutions in Chiba Prefecture and through web-based and newspaper advertisements. As all participants will continue to be treated by their general practitioners, participants will be required to obtain permission from their

For peer review only - http://bmjopen.bmj.com/site/about/guidelines.xhtml

general practitioners prior to study enrolment. This study will be conducted at the academic outpatient clinic of the Cognitive Behavioral Therapy Center of Chiba University Hospital, Japan.

Interventions

UC (conventional treatment)

Participants will be permitted to continue using hypnotics during the study period. There will be no restrictions on treatment options for participants who receive treatments, including medication changes, from their general practitioners. General practitioners will be permitted to refer participants for counseling or to secondary care if deemed clinically appropriate. However, the independent initiation of a strictly structured ICBT program will be prohibited in order to properly assess the effectiveness of the current ICBT. All changes in conventional treatment, along with the reasons for those changes, will be recorded throughout the study period. As part of UC, both UC only and ICBT plus UC groups will receive magazines (in PDF format) developed and sent by email by our research team. Magazines will be sent four times over a 6-week period and will contain information about insomnia and hypnotics which are generally provided by general practitioners. Magazine content will include the following: (1)

For peer review only - http://bmjopen.bmj.com/site/about/guidelines.xhtml

"What you need to know about insomnia" (week 1); (2) "What you need to know about sleeping drugs" (week 3); (3) "How to use sleeping drugs correctly" (week 5); and (4) "How to get off sleeping drugs in the near future" (week 6).

ICBT program

The ICBT program for insomnia was developed by one of the researchers (ES). Treatment consists of five weekly lessons and includes various elements that are commonly incorporated in face-to-face CBT for insomnia. The primary components of this protocol include the following: (1) recording a sleep diary and understanding sleep hygiene; (2) changing sleep-related behaviors, including stimulus control; (3) restructuring distorted beliefs about sleep and sleep-related worries; (4) sleep restriction to increase sleep efficiency; and (5) relaxation training, including breathing exercises and progressive muscle relaxation. Participants will complete the five lessons over a 6-week period to provide sufficient time to become accustomed to CBT.

Every lesson will provide information, examples of other people carrying out the CBT treatment, and homework. One of the researchers (DS) is a cognitive behavioral therapist and will send weekly emails to participants to ask them about their progress. The aim of this email support by a therapist is to allow participants to

For peer review only - http://bmjopen.bmj.com/site/about/guidelines.xhtml

comment on the ICBT exercise, clarify information, and motivate them to persist in carrying out the ICBT program and requested homework. Participants will also be permitted to send emails to DS if they have questions about the content provided. Participants in the control group will also be offered the ICBT treatment after the trial, if the usual care will not have made them sleep better.

Outcomes

Baseline and clinical characteristics

Baseline characteristics will include gender, age, education, marital status, employment status, age at insomnia onset, and duration of insomnia. Moreover, treatment history will include the names of medications to which the participant has exhibited resistance, other prior treatments, current treatment (medication and others) at baseline, and all changes in conventional treatment throughout the study period.

Primary outcome

The primary outcome will be PSQI score at week 6. PSQI is a self-rated questionnaire consisting of 19 questions across seven subscales (sleep quality, sleep latency, sleep duration, habitual sleep efficiency, sleep disturbance, use of hypnotics,

For peer review only - http://bmjopen.bmj.com/site/about/guidelines.xhtml

and daytime dysfunction). Each subscale is scored on a scale of 0–3. Subscale scores are summed to a total score ranging from 0 (good quality of sleep) to 21 (very poor quality of sleep). PSQI was verified as a reliable and valid measure of subjective sleep quality in clinical practice and experimental research (48, 49).

Secondary outcomes

Secondary outcomes will consist of sleep onset latency, total sleep time, and sleep efficiency extracted from PSQI, as well as current feeling of refreshment and perceived soundness of sleep (assessed by visual analog scale), number of awakenings (assessed by HADS) to measure anxiety, CES-D score to measure depression, and Euro Qol-5D (EQ-5D) score to measure QOL.

Total score on the seven HADS anxiety subscale items ranges from 0 (no symptoms of anxiety) to 21 (severe symptoms of anxiety). Total score on the 20 CES-D items ranges from 0 (no symptoms of depression) to 60 (severe symptoms of depression). The 3-level version of EQ-5D was described in our previous study (50). The EQ-5D (50) contains five items that assess QOL on a 3-point Likert scale ranging from 1 (not severe) to 3 (severe). The Japanese version of EQ-5D was developed by Tsuchiya et al. (51). EQ-5D is the most commonly used scale worldwide for calculating

For peer review only - http://bmjopen.bmj.com/site/about/guidelines.xhtml

For peer review only

quality-adjusted life years (QALYs). QALYs are often used as the health outcome in cost–utility analyses and are typically estimated via area under the curve analysis, which involves summing the areas of the distribution shapes to calculate utility scores over the study period (50, 52). In the present study, QALYs will be assessed using EQ-5D, an indicator of patient health status. This index is calculated by transforming the EQ-5D dimension scores into a single summary score ranging from 0 to 1 (1 = full health) by applying a formula created by the EuroQol Group (50, 53). Participants will complete these questionnaires at home.

The therapist will ask participants about adverse event experiences at each assessment. All measures will be assessed at weeks 0 (baseline), 6 (post-intervention), and 12 (follow-up), and outcomes will be analyzed based on the intention-to-treat principle.

Sample size

The projected sample size was based on a previous study by van Straten et al. (45), which indicated that the estimated group difference in changes of PSQI scores from baseline was approximately 2.86 (ICBT group = 3.00; control group = 0.04). The conventional treatment (i.e., UC) alone is assumed to be largely ineffective. Assuming a

For peer review only - http://bmjopen.bmj.com/site/about/guidelines.xhtml

group difference of 2.86 points [standard deviation (SD) = 2.5], 13 subjects per group will provide 80% power to detect a difference in PSQI scores between the UC arm and ICBT plus UC arm for at least 6 weeks, using a two-sided, two-sample t-test at a 5% significance level. Thus, allowing for a 10% dropout rate, 15 participants are required per group, for a total of 30 participants in the study.

Randomization

At the end of the baseline assessment, eligible participants will be randomly assigned to either the UC arm or ICBT plus US arm at a ratio of 1:1, with assignments made using the minimization method, ensuring a balance in baseline PSQI scores (PSQI ≥ 12) and gender.

Each participant will then be assigned to one of the two treatment regimes. Participants will be blinded to the group to which they are assigned before consenting to participate in the study.

Data analysis plan

For peer review only

For peer review only - http://bmjopen.bmj.com/site/about/guidelines.xhtml

Statistical analysis and reporting of this trial will be conducted in accordance with CONSORT guidelines, with primary analyses based on the intention-to-treat principle. For baseline variables, summary statistics will be constructed, employing frequencies and proportions for categorical data and mean and SD for continuous variables. Baseline variables will be compared using the Fisher's exact test for categorical outcomes and the unpaired t-test for continuous variables.

For the primary analysis comparing treatment effects, the least-squares means and their 95% confidence intervals will be estimated by analysis of covariance (ANCOVA) with the change in total PSQI scores at week 6. This ANCOVA model will take into account the variation caused by treatment effects, and gender and baseline PSQI score will be entered as covariates. Analyses of secondary outcomes will be performed in the same manner as the primary analysis.

All comparisons are planned, and all p-values will be two-sided. P-values <0.05 will be considered statistically significant. All statistical analyses will be performed using SAS version 9.4 (SAS Institute, Cary, NC, USA).

For peer review only

For peer review only - http://bmjopen.bmj.com/site/about/guidelines.xhtml

Ethics and dissemination

This study will be conducted at the Academic Outpatient Clinic of the Cognitive Behavioral Therapy Center at Chiba University Hospital, Japan. The study protocol was approved by the Institutional Review Board of the Chiba University Hospital (reference number: G27040) in December 2015.

When potential participants contact the study trial office, they will be informed of the study objectives and asked if they are willing to participate. Each patient will be informed that participation is voluntary and that full anonymity will be provided. Each participant will then be required to provide written informed consent for their participation in this study. Each participant will also be informed that all participants will receive UC (conventional treatment) from their general practitioner and that half of the recruited participants will also receive ICBT in addition to their UC. A physician's examination will be conducted at each assessment point (weeks 0, 6, and 12).

An adverse event could consist of any unfavorable and unintended sign, symptom, or disease temporally associated with this interventional study, whether or not considered related to the ICBT program. All adverse events will be reported, and

For peer review only - http://bmjopen.bmj.com/site/about/guidelines.xhtml

serious adverse events will be immediately reported to the Institutional Review Board of Chiba University Hospital in addition to being registered with the hospital risk management system. Moreover, an independent data monitoring committee will accurately verify detailed records of the clinical study's progress, critical efficacy variables, and safety data, and recommend the sponsor to continue, modify, or terminate the study accordingly.

Study outcomes will be published in international journals, regardless of the outcome. The study will be conducted and reported according to the CONSORT recommendations.

DISCUSSION

This pilot study was planned to address the lack of trials examining the administration of ICBT in patients with insomnia after hypnotic treatment. The findings of this study will provide valuable evidence to facilitate the development of second-line treatments after pharmacotherapy and to establish treatment algorithms. The foreseen limitations of this study are the following. First, sleep estimates will be based on

For peer review only - http://bmjopen.bmj.com/site/about/guidelines.xhtml

subjective sleep diaries and PSQI scores, rather than on objective measures such as polysomnography or actigraphy. The use of both subjective and objective measures has been recommended because patients with insomnia underestimate their actual sleep time (11, 17). However, using polysomnography is costly and imposes significant burden on participants. Therefore, sleep diaries are currently the most widely used outcome measure in insomnia treatment studies (5) and are generally well accepted because subjective complaints generally prompt participants to seek treatment. The second limitation is that we will be unable to elucidate specific effects of the ICBT program because a psychological placebo group to control for nonspecific factors will not be employed. The third limitation is that there is a possibility for improvement of control group as ICBT is provided to the applicant of the control group after the end of the follow-up period.

For peer review only

For peer review only - http://bmjopen.bmj.com/site/about/guidelines.xhtml

References

1. American Psychiatric Association. Diagnostic and statistical manual of mental disorders. 5th ed. Washington: American Psychiatric Association; 2013.

2. Ohayon MM. Epidemiology of insomnia: what we know and what we still need to learn. Sleep Med Rev. 2002; 6: 97-111.

3. Lichstein KL, Durrence HH, Reidel BW, et al. The epidemiology of sleep: age, gender and ethnicity. Mahwah: Lawrence Erlbaum Associates; 2004.

4. Morphy H, Dunn KM, Lewis M, et al. Epidemiology of insomnia: a longitudinal study in a UK Population. Sleep. 2007; 30 (3): 274–80.

5. Morin CM, Belanger L, LeBlanc M, et al. The natural history of insomnia: a population-based 3-year longitudinal study. Arch Intern Med. 2009; 169: 447-53.

6. Baglioni C, Battagliese G, Feige B, et al. Insomnia as a predictor of depression: a meta-analytic evaluation of longitudinal epidemiological studies. J Affect Disord. 2011; 135: 10-9.

7. Vgontzas AN, Liao D, Bixler EO, Chrousos GP, Vela-Bueno A. Insomnia with objective short sleep duration is associated with a high risk for hypertension. Sleep. 2009; 32: 491-7.

For peer review only - http://bmjopen.bmj.com/site/about/guidelines.xhtml

For peer review only

8. Vgontzas AN, Liao D, Pejovic S, Calhoun S, Karataraki M, Bixler EO. Insomnia with objective short sleep duration is associated with type 2 diabetes: a population-based study. Diabetes Care. 2009; 32: 1980-5.

9. Cappuccio FP, D'Elia L, Strazzullo P, Miller MA. Quantity and quality of sleep and incidence of type 2 diabetes. A systematic review and meta-analysis. Diabetes Care. 2010; 33 (2): 414-20.

10. Luyster FS, Strollo PJ, Zee PC, on behalf of the Boards of Directors of the American Academy of Sleep Medicine and the Sleep Research Society JK. Sleep: a health imperative. Sleep. 2012; 35 (6): 727-34.

11. Buysse DJ. Sleep health: can we define it? Does it matter? Sleep. 2014; 37 (1): 9-17.

12. Roth T, Ancoli-Israel S. Daytime consequences of insomnia in the United States: results of the 1991 National Sleep Foundation Survey II. Sleep. 1999; 22 Suppl 2: S354-63.

13. Kyle SD, Morgan K, Espie C. Insomnia and health-related quality of life. Sleep Med Rev. 2010; 14: 69-82.

14. Espie CA, Kyle SD, Hames P, et al. The daytime impact of DSM-5 insomnia disorder: comparative analysis of insomnia subtypes from the Great British Sleep Survey. J Clin Psychiatry. 2012; 73: e1478-84.

For peer review only

15. Stepanski E, Koshorek G, Zorick F, Glinn M, Roehrs T, Roth T. Characteristics of individuals who do or do not seek treatment for chronic insomnia. Psychosomatics. 1989; 30: 421-7.

16. Morin CM, LeBlanc M, Daley M, Gregoire JP, Mérette C. Epidemiology of insomnia: prevalence, self-help treatments, consultations, and determinants of help-seeking behaviors. Sleep Med 2007; 7 (2): 123-30.

17. Buysse DJ, Thompson W, Scott J, Franzen PL, Germain A, Hall M, et al. Daytime symptoms in primary insomnia: a prospective analysis using ecological momentary assessment. Sleep Med 2007; 8 (3): 198-208.

18. Levitt H, Wood A, Moul DE, Hall M, Germain A, Kupfer DJ, et al. A pilot study of subjective daytime alertness and mood in primary insomnia participants using ecological momentary assessment. Behav Sleep Med 2004; 2 (2): 113-31.

19. Morin CM, Benca RM. Chronic insomnia. Lancet. 2012; 379: 1129-41.

20. International Narcotics Control Board. Report of the International Narcotics Control Board on the Availability of Internationally Controlled Drugs: Ensuring Adequate Access for Medical and Scientific Purposes. 2010; 32-41. http://apps.who.int/medicinedocs/en/m/abstract/Js21969en/.

For peer review only

For peer review only - http://bmjopen.bmj.com/site/about/guidelines.xhtml

21. Benca RM. Diagnosis and Treatment of Chronic Insomnia: A Review. Psychiatr Serv 2005; 56: 332-43.

22. National Institutes of Health. National Institutes of Health State of the Science Conference Statement on manifestations and management of chronic insomnia in adults 2005. Sleep 2005; 28: 1049-57.

23. Schutte-Rodin S, Broch L, Buysse D, Dorsey C, Sateia M. Clinical guideline for the evaluation and management of chronic insomnia in adults. J Clin Sleep Med. 2008; 4(5): 487-504.

24. Wilson SJ, Nutt DJ, Alford C, Argyropoulos SV, Baldwin DS, Bateson AN, Britton TC, Crowe C, Dijk DJ, Espie CA, Gringras P, Hajak G, Idzikowski C, Krystal AD, Nash JR, Selsick H, Sharpley AL, Wade AG. British Association for Psychopharmacology consensus statement on evidence-based treatment of insomnia, parasomnias and circadian rhythm disorders. J Psychopharmacol. 2010; 24(11): 1577-601.

25. Qaseem A, Kansagara D, Forciea MA, Cooke M, Denberg TD; Clinical Guidelines Committee of the American College of Physicians. Management of Chronic Insomnia Disorder in Adults: A Clinical Practice Guideline From the American College of Physicians. Ann Intern Med. 2016; 165(2): 125-33.

For peer review only

For peer review only - http://bmjopen.bmj.com/site/about/guidelines.xhtml

26. Riemersma-van der Lek RF, Swaab DF, Twisk J, Hol EM, Hoogendijk WJ, Van Someren EJ. Effect of bright light and melatonin on cognitive and noncognitive function in elderly residents of group care facilities: a randomized controlled trial. JAMA 2008; 299(22): 2642-55.

27. Montgomery P, Dennis J. A systematic review of non-pharmacological therapies for sleep problems in later life. Sleep Med Rev 2004; 8(1): 47-62.

28. Vallières A, Morin CM, Guay B. Sequential combinations of drug and cognitive behavioral therapy for chronic insomnia: an exploratory study. Behav Res Ther. 2005; 43(12): 1611-30.

29. Morin CM, Vallières A, Guay B, Ivers H, Savard J, Mérette C, et al. Cognitive behavioral therapy, singly and combined with medication, for persistent insomnia. JAMA 2009; 301: 2005-15.

30. NIH. State-of-the-Science Conference Statement on Manifestations and Management of Chronic Insomnia in Adults. NIH Consens Sci Statements 2005; 22: 1-30.

31. Taylor DJ, Schmidt-Nowara W, Jessop CA, Ahearn J. Sleep restriction therapy and hypnotic withdrawal versus sleep hygiene education in hypnotic using patients with insomnia. J Clin Sleep Med.2010; 6(2): 169-75.

For peer review only

For peer review only - http://bmjopen.bmj.com/site/about/guidelines.xhtml

32. Baillargeon L, Demers M, Ladouceur R. Stimulus-control: nonpharmacologic treatment for insomnia. Can Fam Physician.1998; 44: 73-9.

33. Spielman AJ, Caruso LS, Glovinsky PB. A behavioral perspective on insomnia treatment. Psychiatr Clin North Am 1987; 10(4): 541-53.

34. Wilson SJ, Nutt DJ, Alford C, et al. British Association for Psychopharmacology consensus statement on evidence-based treatment of insomnia, parasomnias and circadian rhythm disorders. J Psychopharmacol 2010; 24: 1577-600.

35. Trauer JM, Qian MY, Doyle JS, Rajaratnam SMW, Cunnington D. Cognitive behavioral therapy for chronic insomnia: a systematic review and metaanalysis. Ann Intern Med. 2015; 163: 191-204.

36. Wu JQ, Appleman ER, Salazar RD, Ong JC. Cognitive behavioral therapy for insomnia comorbid with psychiatric and medical conditions: a metaanalysis. JAMA Intern Med 2015; 175: 1461-72.

37. Mitchell MD, Gehrman P, Perlis M, Umscheid CA. Comparative effectiveness of cognitive behavioral therapy for insomnia: a systematic review. BMC Fam Pract 2012; 13 (1): 40.

38. Morin CM, Vallières A, Guay B, Ivers H, Savard J, Mérette C, et al. Cognitivebehavior therapy, singly and combined with medication, for persistent

For peer review only - http://bmjopen.bmj.com/site/about/guidelines.xhtml

insomnia: acute and maintenance therapeutic effects. JAMA 2009; 301 (19): 2005-15.

39. Espie CA, Inglis SJ, Tessier S, Harvey L. The clinical effectiveness of cognitive behaviour therapy for chronic insomnia: implementation and evaluation of a sleep clinic in general medical practice. Behav Res Ther. 2001; 39 (1) :45-60.

40. Espie CA, MacMahon KM, Kelly H, et al. Randomized clinical effectiveness trial of nurse-administered small group cognitive behaviour therapy for persistent insomnia in general practice. Sleep 2007; 30: 574-84.

41. Espie CA, Fleming L, Cassidy J, et al. Randomized controlled clinical effectiveness trial of cognitive behaviour therapy compared with treatment as usual for persistent insomnia in patients with cancer. J Clin Oncol 2008; 26: 4651-8.

42. Espie CA, Kyle SD, Miller CB, Ong J, Hames P, Fleming L. Attribution,cognition and psychopathology in persistent insomnia disorder: outcome and mediation analysis from a randomized placebo-controlled trial of online cognitive behavioural therapy. Sleep Med 2014; 15 (8): 913-7.

43. Okajima, I., Nakamura, M., Nishida, S., Usui, A., Kanno, M., Nakajima, S., & Inoue, Y. Cognitive behavioural therapy with behavioural analysis for pharmacological treatment-resistant chronic insomnia. Psychiatry Research 2013; 210(2): 515-521.

For peer review only - http://bmjopen.bmj.com/site/about/guidelines.xhtml

44. Zachariae R, Lyby MS, Ritterband LM, O'Toole MS. Efficacy of internet-delivered cognitive-behavioral therapy for insomnia - A systematic review and meta-analysis of randomized controlled trials. Sleep Med Rev. 2016; 30: 1-10.

45. Van Straten, Emmelkamp, de Wit, Lancee, Andersson, van Someren EJ, Cuijpers P. Guided Internet-delivered cognitive behavioural treatment for insomnia: a randomized trial. Psychol Med 2014; 44(7): 1521-32.

46. Kaldo V, Jernelöv S, Blom K, Ljótsson B, Brodin M, Jörgensen M, Kraepelien M, Rück C, Lindefors N. Guided internet cognitive behavioral therapy for insomnia compared to a control treatment - A randomized trial. Behav Res Ther 2015; 71: 90-100.

47. Yoshinaga N, Niitsu T, Hanaoka H, Sato Y, Ohshima F, Matsuki S, Kobori O, Nakazato M, Nakagawa A, Iyo M, Shimizu E. Strategy for treating selective serotonin reuptake inhibitor-resistant social anxiety disorder in the clinical setting: a randomised controlled trial protocol of cognitive behavioural therapy in combination with conventional treatment. BMJ Open. 2013 Feb 13; 3(2): pii: e002242.

48. Carpentera J, Andrykowskia M. Psychometric evaluation of the pittsburgh sleep quality index. Journal of Psychosomatic Research 1998; 45(1): 5-13.

For peer review only - http://bmjopen.bmj.com/site/about/guidelines.xhtml

49. Doi Y, Minowa M, Uchiyama M, Okawa M, Kim K, Shibui K, Kamei Y. Psychometric assessment of subjective sleep quality using the Japanese version of the Pittsburgh Sleep Quality Index (PSQI-J) in psychiatric disordered and control subjects. Psychiatry Research 2000; 97(2-3): 165-72.

50. Seki Y, Nagata S, Shibuya T, Yoshinaga N, Yokoo M, Ibuki H, Minamitani N, Kusunoki M, Inada Y, Kawasoe N, Adachi S, Yoshimura Y, Nakazato M, Iyo M, Nakagawa A, Shimizu E. A Feasibility Study of the Clinical Effectiveness and Cost-Effectiveness of Individual Cognitive Behavioral Therapy for Panic Disorder in a Japanese Clinical Setting: An Uncontrolled Pilot Study. BMC Research Notes 2016; 9(1): 458.

51. Tsuchiya A, Ikeda S, Ikegami N, Nishimura S, Sakai I, Fukuda T, Hamashima C, Hisashige A, Tamura M. Estimating an EQ-5D population value set: the case of Japan. Health Econ 2002; 11: 341–53.

52. EuroQol Group. A new facility for the measurement of health-related quality of life. Health Policy. 1990; 16: 199–208.

53. Manca A, Hawkins N, Sculpher MJ. Estimating mean QALYs in a trial-based cost-effectiveness analysis: the importance of controlling for baseline utility. Health Econ 2005; 14: 487–96.

For peer review only

For peer review only - http://bmjopen.bmj.com/site/about/guidelines.xhtml

Authors' affiliations

[1]Departments of Cognitive Behavioral Physiology Graduate School of Medicine, Chiba University, Chiba, Japan

[2]Department of Rehabilitation Sciences, Faculty of Health Care Sciences, Chiba Prefectural University of Health Sciences, Chiba, Japan

[3]Organization for Promotion of Tenure Track, University of Miyazaki, Miyazaki, Japan

[4]Clinical Research Center, Chiba University Hospital, Chiba, Japan

[5]Department of Global Clinical Research / Biostatistics, Graduate School of Medicine, Chiba University, Chiba, Japan

[6]Research Center for Child Mental Development, Chiba University, Chiba, Japan

[7]Cognitive Behavioral Therapy Center, Chiba University Hospital, Chiba, Japan

For peer review only

Authors' contributions

Daisuke Sato and Eiji Shimizu designed the study and wrote the initial draft of the manuscript. Yasunori Sato contributed to analysis and interpretation of data and assisted in the preparation of the manuscript. All other authors have contributed to data collection and interpretation and critically reviewed the manuscript. All authors approved the final version of the manuscript and agree to be accountable for all aspects

For peer review only - http://bmjopen.bmj.com/site/about/guidelines.xhtml

of the work in ensuring that questions related to the accuracy or integrity of any part of the work are appropriately investigated and resolved.

Funding statement: None.

Competing interests statement None.

For peer review only

For peer review only - http://bmjopen.bmj.com/site/about/guidelines.xhtml

Figure legends:

Figure 1 CONSORT patients' flow diagram during inclusion, randomization and treatment.

For peer review only

For peer review only - http://bmjopen.bmj.com/site/about/guidelines.xhtml

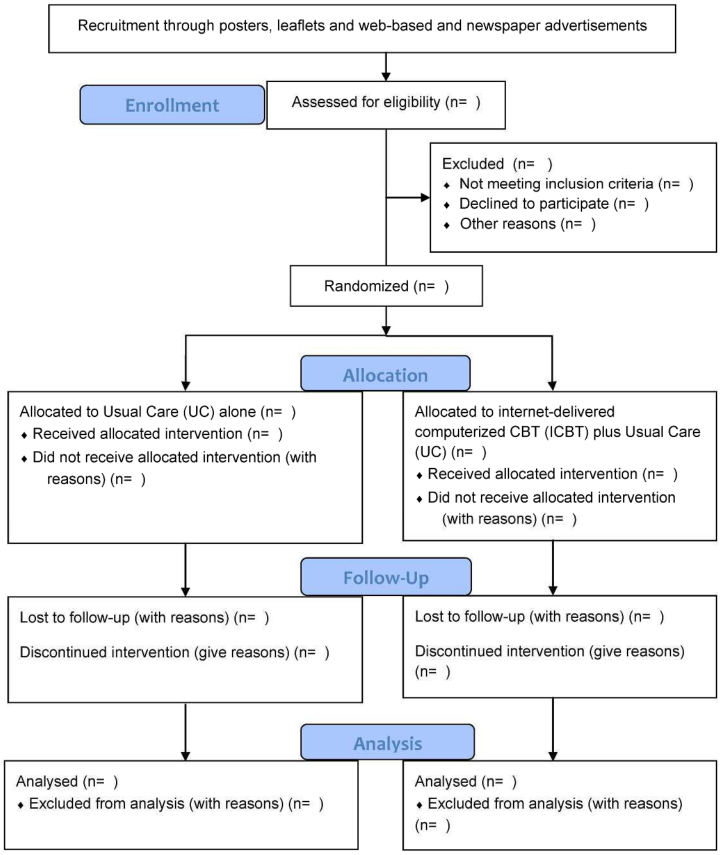

Figure 1 CONSORT patients' flow diagram during inclusion, randomization and treatment.

279x361mm (300 x 300 DPI)

SPIRIT

STANDARD PROTOCOL ITEMS: RECOMMENDATIONS FOR INTERVENTIONAL TRIALS

SPIRIT 2013 Checklist: Recommended items to address in a clinical trial protocol and related documents*

Section/item	Item No	Description	Addressed on page number
**Administrative information**			
Title	1	Descriptive title identifying the study design, population, interventions, and, if applicable, trial acronym	1
Trial registration	2a	Trial identifier and registry name. If not yet registered, name of intended registry	4 https://upload.umin.ac.jp/cgi-open-bin/ctr/ctr_view.cgi?recptno=R000023545
	2b	All items from the World Health Organization Trial Registration Data Set	Yes
Protocol version	3	Date and version identifier	2
Funding	4	Sources and types of financial, material, and other support	34
Roles and responsibilities	5a	Names, affiliations, and roles of protocol contributors	1, 33, 34
	5b	Name and contact information for the trial sponsor	34
	5c	Role of study sponsor and funders, if any, in study design; collection, management, analysis, and interpretation of data; writing of the report; and the decision to submit the report for publication, including whether they will have ultimate authority over any of these activities	34

For peer review only - http://bmjopen.bmj.com/site/about/guidelines.xhtml

	5d	Composition, roles, and responsibilities of the coordinating centre, steering committee, endpoint adjudication committee, data management team, and other individuals or groups overseeing the trial, if applicable (see Item 21a for data monitoring committee)	21, 22
**Introduction**			
Background and rationale	6a	Description of research question and justification for undertaking the trial, including summary of relevant studies (published and unpublished) examining benefits and harms for each intervention	6, 7, 8, 9, 10, 11
	6b	Explanation for choice of comparators	14, 15
Objectives	7	Specific objectives or hypotheses	11
Trial design	8	Description of trial design including type of trial (eg, parallel group, crossover, factorial, single group), allocation ratio, and framework (eg, superiority, equivalence, noninferiority, exploratory)	11, 12
**Methods: Participants, interventions, and outcomes**			
Study setting	9	Description of study settings (eg, community clinic, academic hospital) and list of countries where data will be collected. Reference to where list of study sites can be obtained	13, 14
Eligibility criteria	10	Inclusion and exclusion criteria for participants. If applicable, eligibility criteria for study centres and individuals who will perform the interventions (eg, surgeons, psychotherapists)	12, 13
Interventions	11a	Interventions for each group with sufficient detail to allow replication, including how and when they will be administered	14, 15, 16
	11b	Criteria for discontinuing or modifying allocated interventions for a given trial participant (eg, drug dose change in response to harms, participant request, or improving/worsening disease)	21, 22
	11c	Strategies to improve adherence to intervention protocols, and any procedures for monitoring adherence (eg, drug tablet return, laboratory tests)	15, 16
	11d	Relevant concomitant care and interventions that are permitted or prohibited during the trial	14

For peer review only - http://bmjopen.bmj.com/site/about/guidelines.xhtml

Outcomes	12	Primary, secondary, and other outcomes, including the specific measurement variable (eg, systolic blood pressure), analysis metric (eg, change from baseline, final value, time to event), method of aggregation (eg, median, proportion), and time point for each outcome. Explanation of the clinical relevance of chosen efficacy and harm outcomes is strongly recommended	16, 17, 18
Participant timeline	13	Time schedule of enrolment, interventions (including any run-ins and washouts), assessments, and visits for participants. A schematic diagram is highly recommended (see Figure)	13, 14, 15, 16, 17, 18, Figure 1
Sample size	14	Estimated number of participants needed to achieve study objectives and how it was determined, including clinical and statistical assumptions supporting any sample size calculations	18, 19
Recruitment	15	Strategies for achieving adequate participant enrolment to reach target sample size	13, 14
**Methods: Assignment of interventions (for controlled trials)**			
Allocation:			
Sequence generation	16a	Method of generating the allocation sequence (eg, computer-generated random numbers), and list of any factors for stratification. To reduce predictability of a random sequence, details of any planned restriction (eg, blocking) should be provided in a separate document that is unavailable to those who enrol participants or assign interventions	19
Allocation concealment mechanism	16b	Mechanism of implementing the allocation sequence (eg, central telephone; sequentially numbered, opaque, sealed envelopes), describing any steps to conceal the sequence until interventions are assigned	19
Implementation	16c	Who will generate the allocation sequence, who will enrol participants, and who will assign participants to interventions	19
Blinding (masking)	17a	Who will be blinded after assignment to interventions (eg, trial participants, care providers, outcome assessors, data analysts), and how	14, 15, 16
	17b	If blinded, circumstances under which unblinding is permissible, and procedure for revealing a participant's allocated intervention during the trial	19
**Methods: Data collection, management, and analysis**			

For peer review only - http://bmjopen.bmj.com/site/about/guidelines.xhtml

Data collection methods	18a	Plans for assessment and collection of outcome, baseline, and other trial data, including any related processes to promote data quality (eg, duplicate measurements, training of assessors) and a description of study instruments (eg, questionnaires, laboratory tests) along with their reliability and validity, if known. Reference to where data collection forms can be found, if not in the protocol	16, 17, 18, Figure 1
	18b	Plans to promote participant retention and complete follow-up, including list of any outcome data to be collected for participants who discontinue or deviate from intervention protocols	15, 16, 18
Data management	19	Plans for data entry, coding, security, and storage, including any related processes to promote data quality (eg, double data entry; range checks for data values). Reference to where details of data management procedures can be found, if not in the protocol	22
Statistical methods	20a	Statistical methods for analysing primary and secondary outcomes. Reference to where other details of the statistical analysis plan can be found, if not in the protocol	19, 20
	20b	Methods for any additional analyses (eg, subgroup and adjusted analyses)	19, 20
	20c	Definition of analysis population relating to protocol non-adherence (eg, as randomised analysis), and any statistical methods to handle missing data (eg, multiple imputation)	19, 20
**Methods: Monitoring**			
Data monitoring	21a	Composition of data monitoring committee (DMC); summary of its role and reporting structure; statement of whether it is independent from the sponsor and competing interests; and reference to where further details about its charter can be found, if not in the protocol. Alternatively, an explanation of why a DMC is not needed	21, 22
	21b	Description of any interim analyses and stopping guidelines, including who will have access to these interim results and make the final decision to terminate the trial	21, 22
Harms	22	Plans for collecting, assessing, reporting, and managing solicited and spontaneously reported adverse events and other unintended effects of trial interventions or trial conduct	18, 21, 22
Auditing	23	Frequency and procedures for auditing trial conduct, if any, and whether the process will be independent from investigators and the sponsor	21, 22
**Ethics and dissemination**			

For peer review only - http://bmjopen.bmj.com/site/about/guidelines.xhtml

Research ethics approval	24	Plans for seeking research ethics committee/institutional review board (REC/IRB) approval	21, 22
Protocol amendments	25	Plans for communicating important protocol modifications (eg, changes to eligibility criteria, outcomes, analyses) to relevant parties (eg, investigators, REC/IRBs, trial participants, trial registries, journals, regulators)	21, 22
Consent or assent	26a	Who will obtain informed consent or assent from potential trial participants or authorised surrogates, and how (see Item 32)	21, 22
	26b	Additional consent provisions for collection and use of participant data and biological specimens in ancillary studies, if applicable	21, 22
Confidentiality	27	How personal information about potential and enrolled participants will be collected, shared, and maintained in order to protect confidentiality before, during, and after the trial	22
Declaration of interests	28	Financial and other competing interests for principal investigators for the overall trial and each study site	34
Access to data	29	Statement of who will have access to the final trial dataset, and disclosure of contractual agreements that limit such access for investigators	22
Ancillary and post-trial care	30	Provisions, if any, for ancillary and post-trial care, and for compensation to those who suffer harm from trial participation	18, 21, 22
Dissemination policy	31a	Plans for investigators and sponsor to communicate trial results to participants, healthcare professionals, the public, and other relevant groups (eg, via publication, reporting in results databases, or other data sharing arrangements), including any publication restrictions	22
	31b	Authorship eligibility guidelines and any intended use of professional writers	33, 34
	31c	Plans, if any, for granting public access to the full protocol, participant-level dataset, and statistical code	21, 22
**Appendices**			
Informed consent materials	32	Model consent form and other related documentation given to participants and authorised surrogates	Available on request

For peer review only - http://bmjopen.bmj.com/site/about/guidelines.xhtml

Biological specimens	33	Plans for collection, laboratory evaluation, and storage of biological specimens for genetic or molecular analysis in the current trial and for future use in ancillary studies, if applicable	n/a

*It is strongly recommended that this checklist be read in conjunction with the SPIRIT 2013 Explanation & Elaboration for important clarification on the items. Amendments to the protocol should be tracked and dated. The SPIRIT checklist is copyrighted by the SPIRIT Group under the Creative Commons "Attribution-NonCommercial-NoDerivs 3.0 Unported" license.

For peer review only

BMJ Open

Study protocol for a randomized controlled trial on the effect of internet-delivered computerized cognitive behavioral therapy on patients with insomnia who remain symptomatic following hypnotics

Journal:	*BMJ Open*
Manuscript ID	bmjopen-2017-018220.R2
Article Type:	Protocol
Date Submitted by the Author:	28-Nov-2017

Complete List of Authors:	Sato, Daisuke; Chiba University Graduate School of Medicine, Departments of Cognitive Behavioral Physiology; Chiba Prefectural University of Health Sciences, Rehabilitation Sciences<br>Yoshinaga, Naoki; Miyazaki Daigaku, Organization for Promotion of Tenure Track<br>Nagai, Eichi; Chiba University Hospital, Clinical Research Center<br>Hanaoka, Hideki; Chiba University Hospital, Clinical Research Center<br>Sato, Yasunori; Chiba University Graduate School of Medicine, Department of Global Clinical Research / Biostatistics<br>Shimizu, Eiji; Chiba University, Department of Cognitive Behavioral Physiology, Graduate School of Medicine; Chiba University, Research Center for Child Mental Development

**Primary Subject Heading**:	Mental health
Secondary Subject Heading:	Evidence based practice, Patient-centred medicine, Mental health
Keywords:	Cognitive Behavioral Therapy, Insomnia, Randomized Controlled Trial, Internet

SCHOLARONE™
Manuscripts

Study protocol for a randomized controlled trial on the effect of internet-delivered computerized cognitive behavioral therapy on patients with insomnia who remain symptomatic following hypnotics

Daisuke Sato,[1, 2] Naoki Yoshinaga,[3] Eiichi Nagai,[4] Hideki Hanaoka,[4] Yasunori Sato,[5] Eiji Shimizu[1, 6, 7]

1) Departments of Cognitive Behavioral Physiology, Chiba University Graduate School of Medicine, Chiba, Japan
2) Department of Rehabilitation Sciences, Faculty of Health Care Sciences, Chiba Prefectural University of Health Sciences, Chiba, Japan
3) Organization for Promotion of Tenure Track, University of Miyazaki, Miyazaki, Japan
4) Clinical Research Center, Chiba University Hospital, Chiba, Japan
5) Department of Global Clinical Research / Biostatistics, Graduate School of Medicine, Chiba University, Chiba, Japan
6) Research Center for Child Mental Development, Chiba University, Chiba, Japan
7) Cognitive Behavioral Therapy Center, Chiba University Hospital, Chiba, Japan

Corresponding author

Daisuke Sato,[1, 2]

1) Departments of Cognitive Behavioral Physiology, Chiba University Graduate School of Medicine, Chiba, Japan
2) Department of Rehabilitation Sciences, Faculty of Health Care Sciences, Chiba Prefectural University of Health Sciences, Chiba, Japan

Departments of Cognitive Behavioral Physiology, Chiba University Graduate School of Medicine, Chiba, Japan
1-8-1 Inohana, Chuo-ku, Chiba 260-8670, Japan
Tel: (+81) 43-226-2027, Fax: (+81) 43-226-2028
E-mail: daisu.sato@gmail.com

Keywords: Cognitive Behavioral Therapy, Insomnia, Randomized Controlled Trial, Internet

For peer review only - http://bmjopen.bmj.com/site/about/guidelines.xhtml

Word count: 3591

Number of tables and figures: 1

Protocol version: ver.4.0 (20-Oct-2016)

For peer review only

For peer review only - http://bmjopen.bmj.com/site/about/guidelines.xhtml

ABSTRACT

Introduction: Insomnia has severe consequences for health. Primary care physicians in Japan commonly provide hypnotics, which is far from optimal. The recommended treatment for insomnia is cognitive behavioral therapy (CBT). Access to trained therapists, however, is limited. Rather than face-to-face CBT, several researchers have studied internet-delivered computerized CBT (ICBT). This paper describes the study protocol for a randomized controlled trial (RCT) to evaluate effectiveness and feasibility of our newly developed five-step ICBT as an adjunct to usual care (UC) compared with UC alone for patients with insomnia who remain symptomatic following hypnotics.

Methods and analysis: This proposed exploratory RCT comprises two parallel groups (ICBT + UC and UC alone) consisting of 15 participants each (n = 30) diagnosed with insomnia who remain symptomatic after pharmacotherapy. We aim to evaluate the effectiveness of 6 intervention weeks. The primary outcome of insomnia severity will be the Pittsburgh Sleep Quality Index (PSQI) at week 6. Secondary outcomes include sleep onset latency, total sleep time, sleep efficiency extracted from PSQI, current feeling of refreshment and perceived soundness of sleep measured using visual analog

For peer review only

For peer review only - http://bmjopen.bmj.com/site/about/guidelines.xhtml

scale, number of awakenings, anxiety by Hospital Anxiety and Depression Scale, depression by Center for Epidemiologic Studies Depression Scale, and quality of life by Euro Qol-5D. All measures will be assessed at weeks 0 (baseline), 6 (post-intervention), and 12 (follow-up), and intention-to-treat will be applied. The statistical analysis plan has been developed considering design of field materials.

Ethics and dissemination: This study will be conducted at the academic outpatient clinic of Chiba University Hospital, Japan. Ethics approval was granted by the Institutional Review Board of Chiba University Hospital. All participants will be required to provide written informed consent. The trial will be implemented and reported in accordance with CONSORT recommendations.

Clinical Trial Registration Number: UMIN000021509

For peer review only

For peer review only - http://bmjopen.bmj.com/site/about/guidelines.xhtml

ARTICLE SUMMARY

Strengths and limitations of this study

- This is the first RCT to focus on ICBT as a therapeutic option for insomnia patients who remain symptomatic following hypnotics.
- This study reflects good clinical practice, and its results will contribute to the development of second-line treatments and establish future treatment algorithms.
- A study limitation is the inability to elucidate specific effects of the ICBT program because a psychological placebo group to control for nonspecific factors will not be employed and sleep estimates will not be based on objective measures such as polysomnography or actigraphy.

For peer review only

For peer review only - http://bmjopen.bmj.com/site/about/guidelines.xhtml

INTRODUCTION

Insomnia is characterized by the inability to fall asleep or awakening too early in the morning or during the night, resulting in non-restorative sleep and decreased daytime functioning. Insomnia comprises a symptomatic state of difficulty in sleeping, related to significant daytime effects, experienced three or more times a week for over 3 months (1). Worldwide epidemiological research reports the incidence of insomnia to be 10%–12% (2–4). Although the incidence of insomnia is high, spontaneous improvement of the disorder is low. In one study, insomnia persisted a year later in 74% of participants with insomnia and the disorder persisted for over 3 years in 46% (5). The recently revised Diagnostic and Statistical Manual of Mental Disorders, 5th edition (DSM-5) underlines the need for clinical acknowledgment of sleep disorders (1). This recommendation is supported by studies reporting not only that the incidence of mental and physical health co-morbidities are high but that insomnia is a factor associated with mood disorders (6), cardiovascular disease (7), and hyperglycemia (8, 9). From a population health perspective, sleep is a more important issue than was previously recognized (10, 11).

For peer review only - http://bmjopen.bmj.com/site/about/guidelines.xhtml

In general, insomnia is related to worsening fatigue, work efficiency, and decreased quality of life (QOL) (12–14). In spite of this evidence of decreased productivity resulting from sleep deficits and it being a necessary diagnostic standard for insomnia, comparatively little research has been conducted on its association with QOL. Quite amazingly while insomnia's perceived sleep deficit is few, many people begin asking for help, particularly when insomnia's perceived difficulty on personal functioning such as daytime fatigue become more pronounced. (15, 16). In epidemiological research, 20% of the most frequently cited reasons for visiting a sleep disorder clinic were daytime symptoms of fatigue, psychological suffering, physical discomfort as heartburn, a backflow of acid and food from the stomach into the esophagus after eating, and impaired work efficiency (16). Moreover, studies performed by sleep clinicians at consultations show that patients with insomnia complain of disturbances of mood and cognitive abilities coexisting with severe levels of anxiousness, fatigue, pain, and physical discomfort. Once QOL has been severely impacted, individuals often request a consultation (17, 18).

Pharmacotherapy for insomnia

For peer review only - http://bmjopen.bmj.com/site/about/guidelines.xhtml

Although the causes of insomnia are at present inadequately understood, some of the most common treatments provided by primary physicians are drugs including benzodiazepines, non-benzodiazepines, orexin receptor antagonists, melatonin receptor agonists, and antidepressants (19). Furthermore, international research shows that physicians in Japan prescribe excess doses of benzodiazepine (20). Unfortunately, pharmacotherapy is associated with a high incidence of adverse effects, and examples of side effects include daytime sleepiness and recurrence insomnia (21). For this reason, pharmacotherapy is not recommended (21). In recent years, two alternate types of intervention for patients with insomnia have been experimentally examined. The first is cognitive behavioral therapy (CBT). CBT has similar or even better and more long-lasting outcomes than pharmacological therapy and lacks the negative side effects of pharmacological treatments (22). Clinical practice guidelines suggest CBT, rather than pharmacotherapy, as the initial therapy for patients with insomnia (23–25).The second type of intervention is chronobiological treatment, which enhances regular input into the body's circadian rhythms using bright light (26). It has been reported that compared with CBT, the effect of light therapy on sleep maintenance is lower (27). Vallières A et al. reported that pharmacotherapy prior to the initiation of CBT appears to be less effective than the combined treatment of pharmacotherapy and CBT, followed

For peer review only - http://bmjopen.bmj.com/site/about/guidelines.xhtml

by CBT alone (28). This study also found that early introduction of CBT contributes to maximizing the effect of pharmacotherapy.

CBT for insomnia

CBT, regarded as the first-choice treatment for persistent sleep deficits (29, 2430), is a psychological treatment aiming to correct negative thought patterns and behaviors that influence the persistence of insomnia. CBT is composed of a variety of methods including a behavioral component (sleep restriction therapy, stimulus control therapy, and relaxation) in combination with cognitive (managing anxiety about sleep and intrusive thoughts) and educational (sleep hygiene education) components. Sleep restriction therapy is an approach designed to reduce the time in bed to the actual time of sleep being achieved (31). Stimulus control treatment is behavioral directions to associate the bed with sleep and to restore a consistent sleep rhythm (32). Sleep hygiene education includes health practices such as physical exercise and environmental correlates such as light that may further or disturb sleep (33). Studies examining the effect of CBT in patients with insomnia show that CBT has moderate-to-large effects on sleep efficiency and quality, latency to sleep onset, and time of awakening (34–36). Furthermore, a systemic review concluded that there is low- to moderate-grade evidence

For peer review only - http://bmjopen.bmj.com/site/about/guidelines.xhtml

suggesting that CBT for insomnia is more effective than medication and that approximately 60% of patients receiving CBT respond to therapy and 39% enter remission (37). Further research examining the effect of CBT on the daytime symptoms of patients with insomnia is needed. There is some preliminary evidence that CBT may provide generalized benefits (38–40), as well as some exploratory data from small samples suggesting that CBT for patients with insomnia may reduce anxiety and depression symptoms (41, 42). However, a sufficiently powered, well-planned trial examining the relationship between characteristics of sleep, such as sleep quality and QOL is required. Okajima et al. showed that CBT with behavioral analysis is more effective for insomnia and co-morbid depressive symptoms than conventional treatment and that it promotes an earlier reduction in daily doses of hypnotic medication in patients with chronic insomnia who are resistant to pharmacological treatment (43).

Internet-delivered computerized CBT (ICBT) for insomnia

Recent studies have administered CBT for insomnia as an internet-delivered computerized program (44). The choice of a web application is generally motivated by the observation that online programs are more accessible, have low cost, and can be completed at one’s own time. They are also generally preferred over face-to-face

For peer review only
For peer review only - http://bmjopen.bmj.com/site/about/guidelines.xhtml

interventions because they can be accessed anonymously, are convenient, and can be used anywhere. In a recent trial, Van Straten et al. showed statistically significant effects on sleep quality (Pittsburgh Sleep Quality Index; PSQI), total sleep time, and sleep efficiency in participants receiving ICBT for insomnia compared with a wait-list control group (45). Furthermore, ICBT for hypnotics-resistant insomnia was significantly more effective than the control treatment in reducing insomnia severity (Insomnia Severity Index), use of hypnotics, and improving sleep efficiency (46).

Aims

In summary, this paper describes the study protocol for a randomized controlled trial designed to evaluate the clinical effectiveness of ICBT adjunct to usual care (UC) for patients with insomnia who remain symptomatic after pharmacotherapy, compared with UC alone (47, 48).

METHODS AND ANALYSIS

Study design

For peer review only - http://bmjopen.bmj.com/site/about/guidelines.xhtml

This study was designed as a prospective randomized open-labeled endpoint trial ("PROBE") with two parallel intervention groups consisting of (1) a 6-week treatment regime of UC alone and (2) an ICBT combined with UC. (Figure 1)

Participants

Inclusion criteria for this study include going to bed between 20:00 and 02:00; age, 18–65 years; having a primary diagnosis of insomnia according to the DSM-5; and the aforementioned insomnia remaining symptomatic. "Remaining symptomatic" is defined herein as having insomnia that is at least moderate in severity, based on a PSQI score of >5.5, after the use of hypnotics including non-benzodiazepines, benzodiazepines, melatonin receptor agonists, orexin receptor antagonists, and antidepressants.

Exclusion criteria include severe symptoms of anxiety or depression. Anxiety will be assessed using the anxiety subscale of the Hospital Anxiety and Depression Scale (HADS), which contains seven items. Depression will be assessed using the total score of the Center for Epidemiological Studies Depression scale (CES-D). Participants with a HADS score of ≥10 or a CES-D score of ≥30 will be excluded. Participants with

For peer review only - http://bmjopen.bmj.com/site/about/guidelines.xhtml

psychosis, organic mental disorder, or current high risk of suicide, substance abuse or dependence within the 12 months prior to enrolment, antisocial personality disorder, or unstable medical condition will also be excluded.

Eligibility procedure for participation and diagnosis

Treatment history will be confirmed by the prescribing clinician and by chart review. All participants will be evaluated by two researchers [a psychiatrist (ES) and a therapist (DS)] who will also verify patient diagnosis and eligibility. Validity of the initial diagnosis and eligibility will be discussed. Participants will be reevaluated to cover important missing information based on suggestions derived from the discussion, and final diagnosis and eligibility will be confirmed by the two researchers.

Recruitment

The planned recruitment rate is two participants per month, between March 2016 and January 2018, or until a total of 30 participants are recruited through posters and leaflets placed at medical institutions in Chiba Prefecture and through web-based and newspaper advertisements. As all participants will continue to be treated by their general practitioners, participants will be required to obtain permission from their

For peer review only - http://bmjopen.bmj.com/site/about/guidelines.xhtml

general practitioners prior to study enrolment. This study will be conducted at the academic outpatient clinic of the Cognitive Behavioral Therapy Center of Chiba University Hospital, Japan.

Interventions

UC (conventional treatment)

Participants will be permitted to continue using hypnotics during the study period. There will be no restrictions on treatment options for participants who receive treatments, including medication changes, from their general practitioners. General practitioners will be permitted to refer participants for counseling or to secondary care if deemed clinically appropriate. However, the independent initiation of a strictly structured ICBT program will be prohibited in order to properly assess the effectiveness of the current ICBT. All changes in conventional treatment, along with the reasons for those changes, will be recorded throughout the study period. As part of UC, both UC only and ICBT plus UC groups will receive magazines (in PDF format) developed and sent by email by our research team. Magazines will be sent four times over a 6-week period and will contain information about insomnia and hypnotics which are generally provided by general practitioners. Magazine content will include the following: (1)

For peer review only - http://bmjopen.bmj.com/site/about/guidelines.xhtml

"What you need to know about insomnia" (week 1); (2) "What you need to know about sleeping drugs" (week 3); (3) "How to use sleeping drugs correctly" (week 5); and (4) "How to get off sleeping drugs in the near future" (week 6).

ICBT program

The ICBT program for insomnia was developed by one of the researchers (ES). Treatment consists of five weekly lessons and includes various elements that are commonly incorporated in face-to-face CBT for insomnia. The primary components of this protocol include the following: (1) recording a sleep diary and understanding sleep hygiene; (2) changing sleep-related behaviors, including stimulus control; (3) restructuring distorted beliefs about sleep and sleep-related worries; (4) sleep restriction to increase sleep efficiency; and (5) relaxation training, including breathing exercises and progressive muscle relaxation. Participants will complete the five lessons over a 6-week period to provide sufficient time to become accustomed to CBT.

Every lesson will provide information, examples of other people carrying out the CBT treatment, and homework. One of the researchers (DS) is a cognitive behavioral therapist and will send weekly emails to participants to ask them about their progress. The aim of this email support by a therapist is to allow participants to

For peer review only

comment on the ICBT exercise, clarify information, and motivate them to persist in carrying out the ICBT program and requested homework. Participants will also be permitted to send emails to DS if they have questions about the content provided. Participants in the control group will also be offered the ICBT treatment after the trial, if the usual care will not have made them sleep better.

Outcomes

Baseline and clinical characteristics

Baseline characteristics will include gender, age, education, marital status, employment status, age at insomnia onset, and duration of insomnia. Moreover, treatment history will include the names of medications to which the participant has exhibited resistance, other prior treatments, current treatment (medication and others) at baseline, and all changes in conventional treatment throughout the study period.

Primary outcome

The primary outcome will be PSQI score at week 6. PSQI is a self-rated questionnaire consisting of 19 questions across seven subscales (sleep quality, sleep latency, sleep duration, habitual sleep efficiency, sleep disturbance, use of hypnotics,

For peer review only - http://bmjopen.bmj.com/site/about/guidelines.xhtml

and daytime dysfunction). Each subscale is scored on a scale of 0–3. Subscale scores are summed to a total score ranging from 0 (good quality of sleep) to 21 (very poor quality of sleep). PSQI was verified as a reliable and valid measure of subjective sleep quality in clinical practice and experimental research (49, 50).

Secondary outcomes

Secondary outcomes will consist of sleep onset latency, total sleep time, and sleep efficiency extracted from PSQI, as well as current feeling of refreshment and perceived soundness of sleep (assessed by visual analog scale), number of awakenings (assessed by HADS) to measure anxiety, CES-D score to measure depression, and Euro Qol-5D (EQ-5D) score to measure QOL.

Total score on the seven HADS anxiety subscale items ranges from 0 (no symptoms of anxiety) to 21 (severe symptoms of anxiety). Total score on the 20 CES-D items ranges from 0 (no symptoms of depression) to 60 (severe symptoms of depression). The 3-level version of EQ-5D was described in our previous study (51). The EQ-5D (51) contains five items that assess QOL on a 3-point Likert scale ranging from 1 (not severe) to 3 (severe). The Japanese version of EQ-5D was developed by Tsuchiya et al. (52). EQ-5D is the most commonly used scale worldwide for calculating

For peer review only - http://bmjopen.bmj.com/site/about/guidelines.xhtml

quality-adjusted life years (QALYs). QALYs are often used as the health outcome in cost–utility analyses and are typically estimated via area under the curve analysis, which involves summing the areas of the distribution shapes to calculate utility scores over the study period (51, 53). In the present study, QALYs will be assessed using EQ-5D, an indicator of patient health status. This index is calculated by transforming the EQ-5D dimension scores into a single summary score ranging from 0 to 1 (1 = full health) by applying a formula created by the EuroQol Group (51, 54). Participants will complete these questionnaires at home.

The therapist will ask participants about adverse event experiences at each assessment. All measures will be assessed at weeks 0 (baseline), 6 (post-intervention), and 12 (follow-up), and outcomes will be analyzed based on the intention-to-treat principle.

Sample size

The projected sample size was based on a previous study by van Straten et al. (45), which indicated that the estimated group difference in changes of PSQI scores from baseline was approximately 2.86 (ICBT group = 3.00; control group = 0.04). The conventional treatment (i.e., UC) alone is assumed to be largely ineffective. Assuming a

For peer review only - http://bmjopen.bmj.com/site/about/guidelines.xhtml

group difference of 2.86 points [standard deviation (SD) = 2.5], 13 subjects per group will provide 80% power to detect a difference in PSQI scores between the UC arm and ICBT plus UC arm for at least 6 weeks, using a two-sided, two-sample t-test at a 5% significance level. Thus, allowing for a 10% dropout rate, 15 participants are required per group, for a total of 30 participants in the study.

Randomization

At the end of the baseline assessment, eligible participants will be randomly assigned to either the UC arm or ICBT plus US arm at a ratio of 1:1, with assignments made using the minimization method, ensuring a balance in baseline PSQI scores (PSQI ≥ 12) and gender.

Each participant will then be assigned to one of the two treatment regimes. Participants will be blinded to the group to which they are assigned before consenting to participate in the study.

Data analysis plan

For peer review only - http://bmjopen.bmj.com/site/about/guidelines.xhtml

Statistical analysis and reporting of this trial will be conducted in accordance with CONSORT guidelines, with primary analyses based on the intention-to-treat principle. For baseline variables, summary statistics will be constructed, employing frequencies and proportions for categorical data and mean and SD for continuous variables. Baseline variables will be compared using the Fisher's exact test for categorical outcomes and the unpaired t-test for continuous variables.

For the primary analysis comparing treatment effects, the least-squares means and their 95% confidence intervals will be estimated by analysis of covariance (ANCOVA) with the change in total PSQI scores at week 6. This ANCOVA model will take into account the variation caused by treatment effects, and gender and baseline PSQI score will be entered as covariates. Analyses of secondary outcomes will be performed in the same manner as the primary analysis.

All comparisons are planned, and all p-values will be two-sided. P-values <0.05 will be considered statistically significant. All statistical analyses will be performed using SAS version 9.4 (SAS Institute, Cary, NC, USA).

For peer review only - http://bmjopen.bmj.com/site/about/guidelines.xhtml

Ethics and dissemination

This study will be conducted at the Academic Outpatient Clinic of the Cognitive Behavioral Therapy Center at Chiba University Hospital, Japan. The study protocol was approved by the Institutional Review Board of the Chiba University Hospital (reference number: G27040) in December 2015.

When potential participants contact the study trial office, they will be informed of the study objectives and asked if they are willing to participate. Each patient will be informed that participation is voluntary and that full anonymity will be provided. Each participant will then be required to provide written informed consent for their participation in this study. Each participant will also be informed that all participants will receive UC (conventional treatment) from their general practitioner and that half of the recruited participants will also receive ICBT in addition to their UC. A physician's examination will be conducted at each assessment point (weeks 0, 6, and 12).

An adverse event could consist of any unfavorable and unintended sign, symptom, or disease temporally associated with this interventional study, whether or not considered related to the ICBT program. All adverse events will be reported, and

For peer review only - http://bmjopen.bmj.com/site/about/guidelines.xhtml

serious adverse events will be immediately reported to the Institutional Review Board of Chiba University Hospital in addition to being registered with the hospital risk management system. Moreover, an independent data monitoring committee will accurately verify detailed records of the clinical study's progress, critical efficacy variables, and safety data, and recommend the sponsor to continue, modify, or terminate the study accordingly.

Study outcomes will be published in international journals, regardless of the outcome. The study will be conducted and reported according to the CONSORT recommendations.

DISCUSSION

This study was planned to address the lack of trials examining the administration of ICBT in patients with insomnia after hypnotic treatment. The findings of this study will provide valuable evidence to facilitate the development of second-line treatments after pharmacotherapy and to establish treatment algorithms. The foreseen limitations of this study are the following. First, sleep estimates will be based on

For peer review only - http://bmjopen.bmj.com/site/about/guidelines.xhtml

subjective sleep diaries and PSQI scores, rather than on objective measures such as polysomnography or actigraphy. The use of both subjective and objective measures has been recommended because patients with insomnia underestimate their actual sleep time (11, 17). However, using polysomnography is costly and imposes significant burden on participants. Therefore, sleep diaries are currently the most widely used outcome measure in insomnia treatment studies (5) and are generally well accepted because subjective complaints generally prompt participants to seek treatment. The second limitation is that we will be unable to elucidate specific effects of the ICBT program because a psychological placebo group to control for nonspecific factors will not be employed. The third limitation is that there is a possibility for improvement of control group as ICBT is provided to the applicant of the control group after the end of the follow-up period.

For peer review only

For peer review only - http://bmjopen.bmj.com/site/about/guidelines.xhtml

References

1. American Psychiatric Association. Diagnostic and statistical manual of mental disorders. 5th ed. Washington: American Psychiatric Association; 2013.

2. Ohayon MM. Epidemiology of insomnia: what we know and what we still need to learn. Sleep Med Rev. 2002; 6: 97-111.

3. Lichstein KL, Durrence HH, Reidel BW, et al. The epidemiology of sleep: age, gender and ethnicity. Mahwah: Lawrence Erlbaum Associates; 2004.

4. Morphy H, Dunn KM, Lewis M, et al. Epidemiology of insomnia: a longitudinal study in a UK Population. Sleep. 2007; 30 (3): 274–80.

5. Morin CM, Belanger L, LeBlanc M, et al. The natural history of insomnia: a population-based 3-year longitudinal study. Arch Intern Med. 2009; 169: 447-53.

6. Baglioni C, Battagliese G, Feige B, et al. Insomnia as a predictor of depression: a meta-analytic evaluation of longitudinal epidemiological studies. J Affect Disord. 2011; 135: 10-9.

7. Vgontzas AN, Liao D, Bixler EO, Chrousos GP, Vela-Bueno A. Insomnia with objective short sleep duration is associated with a high risk for hypertension. Sleep. 2009; 32: 491-7.

For peer review only - http://bmjopen.bmj.com/site/about/guidelines.xhtml

8. Vgontzas AN, Liao D, Pejovic S, Calhoun S, Karataraki M, Bixler EO. Insomnia with objective short sleep duration is associated with type 2 diabetes: a population-based study. Diabetes Care. 2009; 32: 1980-5.
9. Cappuccio FP, D'Elia L, Strazzullo P, Miller MA. Quantity and quality of sleep and incidence of type 2 diabetes. A systematic review and meta-analysis. Diabetes Care. 2010; 33 (2): 414-20.
10. Luyster FS, Strollo PJ, Zee PC, on behalf of the Boards of Directors of the American Academy of Sleep Medicine and the Sleep Research Society JK. Sleep: a health imperative. Sleep. 2012; 35 (6): 727-34.
11. Buysse DJ. Sleep health: can we define it? Does it matter? Sleep. 2014; 37 (1): 9-17.
12. Roth T, Ancoli-Israel S. Daytime consequences of insomnia in the United States: results of the 1991 National Sleep Foundation Survey II. Sleep. 1999; 22 Suppl 2: S354-63.
13. Kyle SD, Morgan K, Espie C. Insomnia and health-related quality of life. Sleep Med Rev. 2010; 14: 69-82.
14. Espie CA, Kyle SD, Hames P, et al. The daytime impact of DSM-5 insomnia disorder: comparative analysis of insomnia subtypes from the Great British Sleep Survey. J Clin Psychiatry. 2012; 73: e1478-84.

For peer review only

For peer review only - http://bmjopen.bmj.com/site/about/guidelines.xhtml

15. Stepanski E, Koshorek G, Zorick F, Glinn M, Roehrs T, Roth T. Characteristics of individuals who do or do not seek treatment for chronic insomnia. Psychosomatics. 1989; 30: 421-7.

16. Morin CM, LeBlanc M, Daley M, Gregoire JP, Mérette C. Epidemiology of insomnia: prevalence, self-help treatments, consultations, and determinants of help-seeking behaviors. Sleep Med 2007; 7 (2): 123-30.

17. Buysse DJ, Thompson W, Scott J, Franzen PL, Germain A, Hall M, et al. Daytime symptoms in primary insomnia: a prospective analysis using ecological momentary assessment. Sleep Med 2007; 8 (3): 198-208.

18. Levitt H, Wood A, Moul DE, Hall M, Germain A, Kupfer DJ, et al. A pilot study of subjective daytime alertness and mood in primary insomnia participants using ecological momentary assessment. Behav Sleep Med 2004; 2 (2): 113-31.

19. Morin CM, Benca RM. Chronic insomnia. Lancet. 2012; 379: 1129-41.

20. International Narcotics Control Board. Report of the International Narcotics Control Board on the Availability of Internationally Controlled Drugs: Ensuring Adequate Access for Medical and Scientific Purposes. 2010; 32-41. http://apps.who.int/medicinedocs/en/m/abstract/Js21969en/.

For peer review only

21. Benca RM. Diagnosis and Treatment of Chronic Insomnia: A Review. Psychiatr Serv 2005; 56: 332-43.

22. National Institutes of Health. National Institutes of Health State of the Science Conference Statement on manifestations and management of chronic insomnia in adults 2005. Sleep 2005; 28: 1049-57.

23. Schutte-Rodin S, Broch L, Buysse D, Dorsey C, Sateia M. Clinical guideline for the evaluation and management of chronic insomnia in adults. J Clin Sleep Med. 2008; 4(5): 487-504.

24. Wilson SJ, Nutt DJ, Alford C, Argyropoulos SV, Baldwin DS, Bateson AN, Britton TC, Crowe C, Dijk DJ, Espie CA, Gringras P, Hajak G, Idzikowski C, Krystal AD, Nash JR, Selsick H, Sharpley AL, Wade AG. British Association for Psychopharmacology consensus statement on evidence-based treatment of insomnia, parasomnias and circadian rhythm disorders. J Psychopharmacol. 2010; 24(11): 1577-601.

25. Qaseem A, Kansagara D, Forciea MA, Cooke M, Denberg TD; Clinical Guidelines Committee of the American College of Physicians. Management of Chronic Insomnia Disorder in Adults: A Clinical Practice Guideline From the American College of Physicians. Ann Intern Med. 2016; 165(2): 125-33.

For peer review only

For peer review only - http://bmjopen.bmj.com/site/about/guidelines.xhtml

26. Riemersma-van der Lek RF, Swaab DF, Twisk J, Hol EM, Hoogendijk WJ, Van Someren EJ. Effect of bright light and melatonin on cognitive and noncognitive function in elderly residents of group care facilities: a randomized controlled trial. JAMA 2008; 299(22): 2642-55.

27. Montgomery P, Dennis J. A systematic review of non-pharmacological therapies for sleep problems in later life. Sleep Med Rev 2004; 8(1): 47-62.

28. Vallières A, Morin CM, Guay B. Sequential combinations of drug and cognitive behavioral therapy for chronic insomnia: an exploratory study. Behav Res Ther. 2005; 43(12): 1611-30.

29. Morin CM, Vallières A, Guay B, Ivers H, Savard J, Mérette C, et al. Cognitive behavioral therapy, singly and combined with medication, for persistent insomnia. JAMA 2009; 301: 2005-15.

30. NIH. State-of-the-Science Conference Statement on Manifestations and Management of Chronic Insomnia in Adults. NIH Consens Sci Statements 2005; 22: 1-30.

31. Taylor DJ, Schmidt-Nowara W, Jessop CA, Ahearn J. Sleep restriction therapy and hypnotic withdrawal versus sleep hygiene education in hypnotic using patients with insomnia. J Clin Sleep Med.2010; 6(2): 169-75.

For peer review only - http://bmjopen.bmj.com/site/about/guidelines.xhtml

32. Baillargeon L, Demers M, Ladouceur R. Stimulus-control: nonpharmacologic treatment for insomnia. Can Fam Physician.1998; 44: 73-9.

33. Spielman AJ, Caruso LS, Glovinsky PB. A behavioral perspective on insomnia treatment. Psychiatr Clin North Am 1987; 10(4): 541-53.

34. Wilson SJ, Nutt DJ, Alford C, et al. British Association for Psychopharmacology consensus statement on evidence-based treatment of insomnia, parasomnias and circadian rhythm disorders. J Psychopharmacol 2010; 24: 1577-600.

35. Trauer JM, Qian MY, Doyle JS, Rajaratnam SMW, Cunnington D. Cognitive behavioral therapy for chronic insomnia: a systematic review and metaanalysis. Ann Intern Med. 2015; 163: 191-204.

36. Wu JQ, Appleman ER, Salazar RD, Ong JC. Cognitive behavioral therapy for insomnia comorbid with psychiatric and medical conditions: a metaanalysis. JAMA Intern Med 2015; 175: 1461-72.

37. Mitchell MD, Gehrman P, Perlis M, Umscheid CA. Comparative effectiveness of cognitive behavioral therapy for insomnia: a systematic review. BMC Fam Pract 2012; 13 (1): 40.

38. Morin CM, Vallières A, Guay B, Ivers H, Savard J, Mérette C, et al. Cognitivebehavior therapy, singly and combined with medication, for persistent

For peer review only - http://bmjopen.bmj.com/site/about/guidelines.xhtml

insomnia: acute and maintenance therapeutic effects. JAMA 2009; 301 (19): 2005-15.

39. Espie CA, Inglis SJ, Tessier S, Harvey L. The clinical effectiveness of cognitive behaviour therapy for chronic insomnia: implementation and evaluation of a sleep clinic in general medical practice. Behav Res Ther. 2001; 39 (1) :45-60.

40. Espie CA, MacMahon KM, Kelly H, et al. Randomized clinical effectiveness trial of nurse-administered small group cognitive behaviour therapy for persistent insomnia in general practice. Sleep 2007; 30: 574-84.

41. Espie CA, Fleming L, Cassidy J, et al. Randomized controlled clinical effectiveness trial of cognitive behaviour therapy compared with treatment as usual for persistent insomnia in patients with cancer. J Clin Oncol 2008; 26: 4651-8.

42. Espie CA, Kyle SD, Miller CB, Ong J, Hames P, Fleming L. Attribution,cognition and psychopathology in persistent insomnia disorder: outcome and mediation analysis from a randomized placebo-controlled trial of online cognitive behavioural therapy. Sleep Med 2014; 15 (8): 913-7.

43. Okajima, I., Nakamura, M., Nishida, S., Usui, A., Kanno, M., Nakajima, S., & Inoue, Y. Cognitive behavioural therapy with behavioural analysis for pharmacological treatment-resistant chronic insomnia. Psychiatry Research 2013; 210(2): 515-521.

For peer review only

44. Zachariae R, Lyby MS, Ritterband LM, O'Toole MS. Efficacy of internet-delivered cognitive-behavioral therapy for insomnia - A systematic review and meta-analysis of randomized controlled trials. Sleep Med Rev. 2016; 30: 1-10.

45. Van Straten, Emmelkamp, de Wit, Lancee, Andersson, van Someren EJ, Cuijpers P. Guided Internet-delivered cognitive behavioural treatment for insomnia: a randomized trial. Psychol Med 2014; 44(7): 1521-32.

46. Kaldo V, Jernelöv S, Blom K, Ljótsson B, Brodin M, Jörgensen M, Kraepelien M, Rück C, Lindefors N. Guided internet cognitive behavioral therapy for insomnia compared to a control treatment - A randomized trial. Behav Res Ther 2015; 71: 90-100.

47. Yoshinaga N, Niitsu T, Hanaoka H, Sato Y, Ohshima F, Matsuki S, Kobori O, Nakazato M, Nakagawa A, Iyo M, Shimizu E. Strategy for treating selective serotonin reuptake inhibitor-resistant social anxiety disorder in the clinical setting: a randomised controlled trial protocol of cognitive behavioural therapy in combination with conventional treatment. BMJ Open. 2013 Feb 13; 3(2): pii: e002242.

48. Yoshinaga N, Matsuki S, Niitsu T, Sato Y, Tanaka M, Ibuki H, Takanashi R, Ohshiro K, Ohshima F, Asano K, Kobori O, Yoshimura K, Hirano Y, Sawaguchi K, Koshizaka M, Hanaoka H, Nakagawa A, Nakazato M, Iyo M, Shimizu E. Cognitive

For peer review only - http://bmjopen.bmj.com/site/about/guidelines.xhtml

Behavioral Therapy for Patients with Social Anxiety Disorder Who Remain Symptomatic following Antidepressant Treatment: A Randomized, Assessor-Blinded, Controlled Trial. Psychother Psychosom. 2016; 85(4): 208-17.

49. Carpentera J, Andrykowskia M. Psychometric evaluation of the pittsburgh sleep quality index. Journal of Psychosomatic Research 1998; 45(1): 5-13.

50. Doi Y, Minowa M, Uchiyama M, Okawa M, Kim K, Shibui K, Kamei Y. Psychometric assessment of subjective sleep quality using the Japanese version of the Pittsburgh Sleep Quality Index (PSQI-J) in psychiatric disordered and control subjects. Psychiatry Research 2000; 97(2-3): 165-72.

51. Seki Y, Nagata S, Shibuya T, Yoshinaga N, Yokoo M, Ibuki H, Minamitani N, Kusunoki M, Inada Y, Kawasoe N, Adachi S, Yoshimura Y, Nakazato M, Iyo M, Nakagawa A, Shimizu E. A Feasibility Study of the Clinical Effectiveness and Cost-Effectiveness of Individual Cognitive Behavioral Therapy for Panic Disorder in a Japanese Clinical Setting: An Uncontrolled Pilot Study. BMC Research Notes 2016; 9(1): 458.

52. Tsuchiya A, Ikeda S, Ikegami N, Nishimura S, Sakai I, Fukuda T, Hamashima C, Hisashige A, Tamura M. Estimating an EQ-5D population value set: the case of Japan. Health Econ 2002; 11: 341–53.

For peer review only - http://bmjopen.bmj.com/site/about/guidelines.xhtml

53. EuroQol Group. A new facility for the measurement of health-related quality of life. Health Policy. 1990; 16: 199–208.

54. Manca A, Hawkins N, Sculpher MJ. Estimating mean QALYs in a trial-based cost-effectiveness analysis: the importance of controlling for baseline utility. Health Econ 2005; 14: 487–96.

For peer review only

Authors' affiliations

[1]Departments of Cognitive Behavioral Physiology Graduate School of Medicine, Chiba University, Chiba, Japan

[2]Department of Rehabilitation Sciences, Faculty of Health Care Sciences, Chiba Prefectural University of Health Sciences, Chiba, Japan

[3]Organization for Promotion of Tenure Track, University of Miyazaki, Miyazaki, Japan

[4]Clinical Research Center, Chiba University Hospital, Chiba, Japan

[5]Department of Global Clinical Research / Biostatistics, Graduate School of Medicine, Chiba University, Chiba, Japan

[6]Research Center for Child Mental Development, Chiba University, Chiba, Japan

[7]Cognitive Behavioral Therapy Center, Chiba University Hospital, Chiba, Japan

For peer review only

Authors' contributions

Daisuke Sato and Eiji Shimizu especially made contribution to the design of this study, development of the original study protocol, and drafting the initial manuscript. Yasunori Sato contributed to develop the statistical analysis plan and assisted in the preparation of the manuscript. Naoki Yoshinaga, Eiichi Nagai, and Hideki Hanaoka contributed to the conceptualization and design of this study and the critical revision of the article for important intellectual content. All authors approved the final version of the manuscript

For peer review only - http://bmjopen.bmj.com/site/about/guidelines.xhtml

and agree to be accountable for all aspects of the work in ensuring that questions related to the accuracy or integrity of any part of the work are appropriately resolved.

Funding statement: None.

Competing interests statement None.

For peer review only

For peer review only - http://bmjopen.bmj.com/site/about/guidelines.xhtml

Figure legends:

Figure 1 CONSORT patients' flow diagram during inclusion, randomization and treatment.

For peer review only

For peer review only - http://bmjopen.bmj.com/site/about/guidelines.xhtml

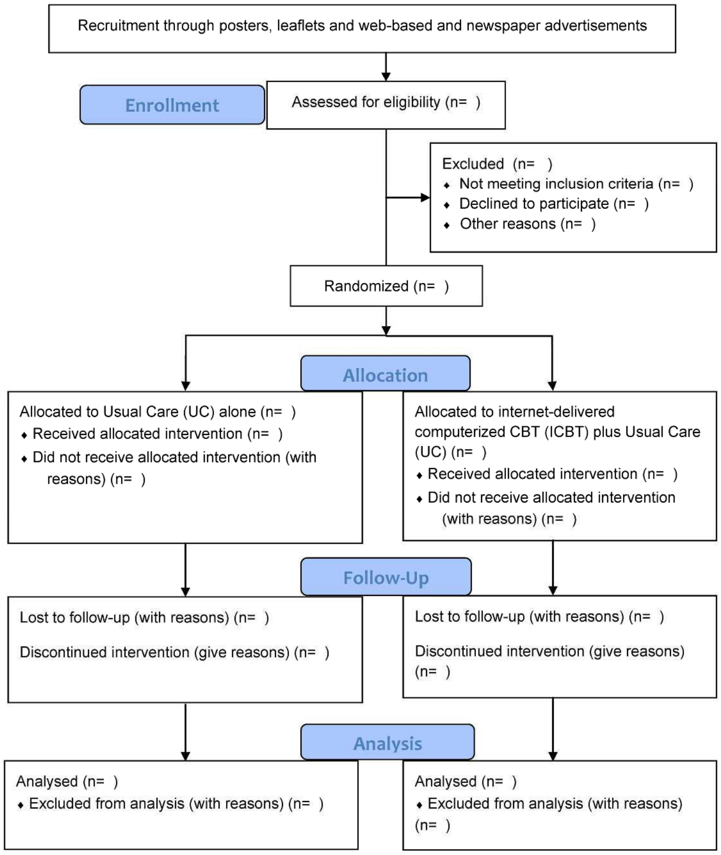

Figure 1 CONSORT patients' flow diagram during inclusion, randomization and treatment.

279x361mm (300 x 300 DPI)

SPIRIT

STANDARD PROTOCOL ITEMS: RECOMMENDATIONS FOR INTERVENTIONAL TRIALS

SPIRIT 2013 Checklist: Recommended items to address in a clinical trial protocol and related documents*

**Section/item**	**Item No**	**Description**	**Addressed on page number**
**Administrative information**			
Title	1	Descriptive title identifying the study design, population, interventions, and, if applicable, trial acronym	1
Trial registration	2a	Trial identifier and registry name. If not yet registered, name of intended registry	4 https://upload.umin.ac.jp/cgi-open-bin/ctr/ctr_view.cgi?recptno=R000023545
	2b	All items from the World Health Organization Trial Registration Data Set	Yes
Protocol version	3	Date and version identifier	2
Funding	4	Sources and types of financial, material, and other support	34
Roles and responsibilities	5a	Names, affiliations, and roles of protocol contributors	1, 33, 34
	5b	Name and contact information for the trial sponsor	34
	5c	Role of study sponsor and funders, if any, in study design; collection, management, analysis, and interpretation of data; writing of the report; and the decision to submit the report for publication, including whether they will have ultimate authority over any of these activities	34

For peer review only

For peer review only - http://bmjopen.bmj.com/site/about/guidelines.xhtml

	5d	Composition, roles, and responsibilities of the coordinating centre, steering committee, endpoint adjudication committee, data management team, and other individuals or groups overseeing the trial, if applicable (see Item 21a for data monitoring committee)	21, 22
**Introduction**			
Background and rationale	6a	Description of research question and justification for undertaking the trial, including summary of relevant studies (published and unpublished) examining benefits and harms for each intervention	6, 7, 8, 9, 10, 11
	6b	Explanation for choice of comparators	14, 15
Objectives	7	Specific objectives or hypotheses	11
Trial design	8	Description of trial design including type of trial (eg, parallel group, crossover, factorial, single group), allocation ratio, and framework (eg, superiority, equivalence, noninferiority, exploratory)	11, 12
**Methods: Participants, interventions, and outcomes**			
Study setting	9	Description of study settings (eg, community clinic, academic hospital) and list of countries where data will be collected. Reference to where list of study sites can be obtained	13, 14
Eligibility criteria	10	Inclusion and exclusion criteria for participants. If applicable, eligibility criteria for study centres and individuals who will perform the interventions (eg, surgeons, psychotherapists)	12, 13
Interventions	11a	Interventions for each group with sufficient detail to allow replication, including how and when they will be administered	14, 15, 16
	11b	Criteria for discontinuing or modifying allocated interventions for a given trial participant (eg, drug dose change in response to harms, participant request, or improving/worsening disease)	21, 22
	11c	Strategies to improve adherence to intervention protocols, and any procedures for monitoring adherence (eg, drug tablet return, laboratory tests)	15, 16
	11d	Relevant concomitant care and interventions that are permitted or prohibited during the trial	14

For peer review only - http://bmjopen.bmj.com/site/about/guidelines.xhtml

Outcomes	12	Primary, secondary, and other outcomes, including the specific measurement variable (eg, systolic blood pressure), analysis metric (eg, change from baseline, final value, time to event), method of aggregation (eg, median, proportion), and time point for each outcome. Explanation of the clinical relevance of chosen efficacy and harm outcomes is strongly recommended	16, 17, 18
Participant timeline	13	Time schedule of enrolment, interventions (including any run-ins and washouts), assessments, and visits for participants. A schematic diagram is highly recommended (see Figure)	13, 14, 15, 16, 17, 18, Figure 1
Sample size	14	Estimated number of participants needed to achieve study objectives and how it was determined, including clinical and statistical assumptions supporting any sample size calculations	18, 19
Recruitment	15	Strategies for achieving adequate participant enrolment to reach target sample size	13, 14
**Methods: Assignment of interventions (for controlled trials)**			
Allocation:			
Sequence generation	16a	Method of generating the allocation sequence (eg, computer-generated random numbers), and list of any factors for stratification. To reduce predictability of a random sequence, details of any planned restriction (eg, blocking) should be provided in a separate document that is unavailable to those who enrol participants or assign interventions	19
Allocation concealment mechanism	16b	Mechanism of implementing the allocation sequence (eg, central telephone; sequentially numbered, opaque, sealed envelopes), describing any steps to conceal the sequence until interventions are assigned	19
Implementation	16c	Who will generate the allocation sequence, who will enrol participants, and who will assign participants to interventions	19
Blinding (masking)	17a	Who will be blinded after assignment to interventions (eg, trial participants, care providers, outcome assessors, data analysts), and how	14, 15, 16
	17b	If blinded, circumstances under which unblinding is permissible, and procedure for revealing a participant's allocated intervention during the trial	19
**Methods: Data collection, management, and analysis**			

Data collection methods	18a	Plans for assessment and collection of outcome, baseline, and other trial data, including any related processes to promote data quality (eg, duplicate measurements, training of assessors) and a description of study instruments (eg, questionnaires, laboratory tests) along with their reliability and validity, if known. Reference to where data collection forms can be found, if not in the protocol	16, 17, 18, Figure 1
	18b	Plans to promote participant retention and complete follow-up, including list of any outcome data to be collected for participants who discontinue or deviate from intervention protocols	15, 16, 18
Data management	19	Plans for data entry, coding, security, and storage, including any related processes to promote data quality (eg, double data entry; range checks for data values). Reference to where details of data management procedures can be found, if not in the protocol	22
Statistical methods	20a	Statistical methods for analysing primary and secondary outcomes. Reference to where other details of the statistical analysis plan can be found, if not in the protocol	19, 20
	20b	Methods for any additional analyses (eg, subgroup and adjusted analyses)	19, 20
	20c	Definition of analysis population relating to protocol non-adherence (eg, as randomised analysis), and any statistical methods to handle missing data (eg, multiple imputation)	19, 20
**Methods: Monitoring**			
Data monitoring	21a	Composition of data monitoring committee (DMC); summary of its role and reporting structure; statement of whether it is independent from the sponsor and competing interests; and reference to where further details about its charter can be found, if not in the protocol. Alternatively, an explanation of why a DMC is not needed	21, 22
	21b	Description of any interim analyses and stopping guidelines, including who will have access to these interim results and make the final decision to terminate the trial	21, 22
Harms	22	Plans for collecting, assessing, reporting, and managing solicited and spontaneously reported adverse events and other unintended effects of trial interventions or trial conduct	18, 21, 22
Auditing	23	Frequency and procedures for auditing trial conduct, if any, and whether the process will be independent from investigators and the sponsor	21, 22
**Ethics and dissemination**			

For peer review only - http://bmjopen.bmj.com/site/about/guidelines.xhtml

Research ethics approval	24	Plans for seeking research ethics committee/institutional review board (REC/IRB) approval	21, 22
Protocol amendments	25	Plans for communicating important protocol modifications (eg, changes to eligibility criteria, outcomes, analyses) to relevant parties (eg, investigators, REC/IRBs, trial participants, trial registries, journals, regulators)	21, 22
Consent or assent	26a	Who will obtain informed consent or assent from potential trial participants or authorised surrogates, and how (see Item 32)	21, 22
	26b	Additional consent provisions for collection and use of participant data and biological specimens in ancillary studies, if applicable	21, 22
Confidentiality	27	How personal information about potential and enrolled participants will be collected, shared, and maintained in order to protect confidentiality before, during, and after the trial	22
Declaration of interests	28	Financial and other competing interests for principal investigators for the overall trial and each study site	34
Access to data	29	Statement of who will have access to the final trial dataset, and disclosure of contractual agreements that limit such access for investigators	22
Ancillary and post-trial care	30	Provisions, if any, for ancillary and post-trial care, and for compensation to those who suffer harm from trial participation	18, 21, 22
Dissemination policy	31a	Plans for investigators and sponsor to communicate trial results to participants, healthcare professionals, the public, and other relevant groups (eg, via publication, reporting in results databases, or other data sharing arrangements), including any publication restrictions	22
	31b	Authorship eligibility guidelines and any intended use of professional writers	33, 34
	31c	Plans, if any, for granting public access to the full protocol, participant-level dataset, and statistical code	21, 22
**Appendices**			
Informed consent materials	32	Model consent form and other related documentation given to participants and authorised surrogates	Available on request

For peer review only

Biological specimens	33	Plans for collection, laboratory evaluation, and storage of biological specimens for genetic or molecular analysis in the current trial and for future use in ancillary studies, if applicable	n/a

*It is strongly recommended that this checklist be read in conjunction with the SPIRIT 2013 Explanation & Elaboration for important clarification on the items. Amendments to the protocol should be tracked and dated. The SPIRIT checklist is copyrighted by the SPIRIT Group under the Creative Commons "Attribution-NonCommercial-NoDerivs 3.0 Unported" license.

For peer review only

For peer review only - http://bmjopen.bmj.com/site/about/guidelines.xhtml
